# Simultaneous priming of HIV broadly neutralizing antibody precursors to multiple epitopes by germline-targeting mRNA-LNP immunogens in mouse models

**DOI:** 10.1126/sciimmunol.adu7961

**Published:** 2025-10-17

**Authors:** Zhenfei Xie, Xuesong Wang, Yu Yan, Jon M. Steichen, Krystal M. Ma, Christopher A. Cottrell, Eleonora Melzi, Maria Bottermann, Paula Maldonado Villavicencio, Kimmo Rantalainen, Torben Schiffner, John E. Warner, Stephanie R. Weldon, Thavaleak Prum, Jordan R. Ellis-Pugh, Jonathan L. Torres, Abigail M. Jackson, Claudia T. Flynn, Gabriel Ozorowski, Sunny Himansu, Andrea Carfi, Andrew B. Ward, Usha Nair, William R. Schief, Facundo D. Batista

**Affiliations:** 1 Batista Lab, The Ragon Institute of Mass General, MIT, and Harvard, Cambridge, MA 02139, USA.; 2 Department of Immunology and Microbiology, The Scripps Research Institute, La Jolla, CA 92037, USA.; 3 International AIDS Vaccine Initiative (IAVI) Neutralizing Antibody Center, The Scripps Research Institute, La Jolla, CA 92037, USA.; 4 Center for HIV/AIDS Vaccine Development, The Scripps Research Institute, La Jolla, CA 92037, USA.; 5 Institute for Drug Discovery, Leipzig University Medical Faculty, Leipzig; 04103, Germany; 6 Department of Integrative Structural and Computational Biology, The Scripps Research Institute, La Jolla, CA 92037, USA.; 7 Moderna Inc., Cambridge, MA 02142, USA.; 8 Department of Biology, Massachusetts Institute of Technology, Cambridge, MA 02139, USA.; 9 Department of Immunology, Harvard Medical School, Boston, MA 02115, USA

**Keywords:** V3-glycan, V2-apex, CD4bs, MPER, germline-targeting, HIV, mRNA-LNP, bnAbs, multiple priming

## Abstract

Germline-targeting is a promising approach to HIV vaccine development that begins with the elicitation of precursors to broadly neutralizing antibodies (bnAbs), but it remains unclear if simultaneous elicitation of precursors to multiple epitopes on the HIV Envelope (Env) would be inhibited by competition. This study used preclinical mouse models with physiologically relevant frequencies of bnAb-precursor-bearing B cells to compare precursor elicitation by co-administration of multiple protein or mRNA lipid nanoparticle (mRNA-LNP) germline-targeting immunogens. These immunogens activate multiple bnAb-precursor classes targeting distinct epitopes on Env, but with evidence of potential competition. Simultaneous delivery of immunogens encoded by mRNA-LNPs, however, drove maturation across different precursor frequencies and immunogen doses. Furthermore, administration of a cocktail of mRNA-LNP immunogens (N332-GT5 gp151, ApexGT5 gp151, eOD-GT8 60mer, and 10E8-GT12 24mer) led to balanced activation of four distinct bnAb-precursor classes, indicating that multi-epitope HIV bnAb-precursor priming might be successfully implemented in humans but might be immunogen dependent.

## Introduction

HIV remains incurable despite the remarkable impact of antiretroviral therapy (ART) (1–4), and approximately 1.7 million people were newly infected in 2019 alone (5). The development of an effective vaccine, therefore, remains an urgent need. The Envelope (Env) spike protein is targeted by most broadly neutralizing antibodies (bnAb) from human HIV patients (6, 7), making it the focal point for vaccine design. However, immunization with wild-type Env has not yet been proven to elicit bnAbs effectively, in part because the unmutated B cell receptors (BCRs) of B cells that may give rise to bnAb-producing descendants do not recognize the epitopes bound by their mature counterparts (8). This gap between germline and mature bnAbs led to the development of germline targeting (GT) vaccine design (9–11). The principle of GT vaccine design is to develop priming immunogens that activate naive B cells bearing germline BCRs that are potential bnAb precursors, which can then undergo somatic hypermutation (SHM) and diversification. Immunization with subsequent immunogens that more closely resemble native Env structures are used to shepherd this pool of activated B cell precursors along a mature-bnAb-like path (9, 12–21). GT immunogens have been developed to elicit different bnAb classes with distinct epitopes: VRC01-class bnAbs to the CD4 binding site (CD4bs) (9, 10, 12, 17, 18, 22, 23), BG18-class bnAbs to the V3-glycan (14, 21, 24), PCT64-class and PG9-class bnAbs to the V2-apex epitope (25–27), and 10E8-class bnAbs to the membrane-proximal external region (MPER) (28, 29). These GT immunogens have demonstrated effective activation of germline precursors, laying the foundation for sequential immunization.

Unfortunately, the genetic diversity of HIV and the dense glycosylation of Env contribute to rapid neutralization escape (30–32) and pose an enormous challenge to Env-based vaccination. Some studies on passive administration of monoclonal antibodies (mAbs) suggest that cocktails of mAbs targeting different sites on Env may be more effective than single-mAb therapies, potentially limiting escape mutation routes (33–39). Effective vaccination may similarly require eliciting more than one class of bnAb targeting more than one site on Env. It is unknown, however, whether bnAb precursors to different epitopes can be primed simultaneously without competition that attenuates the responses, and the logic of GT design ensures that most GT priming immunogens generally elicit site-specific responses (40). As multiple immunizations are already required for bnAb development (13, 15), limiting total injections and healthcare visits by delivering a cocktail of immunogens with each vaccination will likely be key to uptake (41). Thus, the feasibility of GT immunogen coadministration is essential to the overall development of a GT vaccine for HIV.

Preclinical mouse models bearing inferred or genuine germline BCR sequences of human bnAbs provide an effective tool to evaluate pre-clinical vaccine candidates (13–15, 20, 21, 23, 25, 26, 29, 42–48). Previously, we established mouse models with either pre-rearranged heavy only or heavy and light chains from several classes of identified bnAb precursors (25, 49, 50). In this approach, B cells bearing knock-in BCRs are adoptively transferred from CD45.2 hosts to wild-type CD45.1 recipients to create stringent, low-frequency models. Here, we leveraged the adoptive transfer step to introduce different bnAb precursors targeting different epitopes on Env (CD4bs, V3-glycan, V2-apex, or MPER) into the same animal. We then simultaneously delivered two or more previously characterized GT priming immunogens and demonstrated that up to four precursor lineages can enter germinal centers (GCs), remain in those GCs for an extended period, and undergo SHM. This proof-of-principle for priming multiple bnAb classes provides critical insights toward the development of GT HIV vaccines.

## Results

### Co-administration of protein trimer immunogens targeting BG18-class/V3-glycan and PCT64-class/V2-apex precursors efficiently activate BG18-class precursors

**Figure 1.**
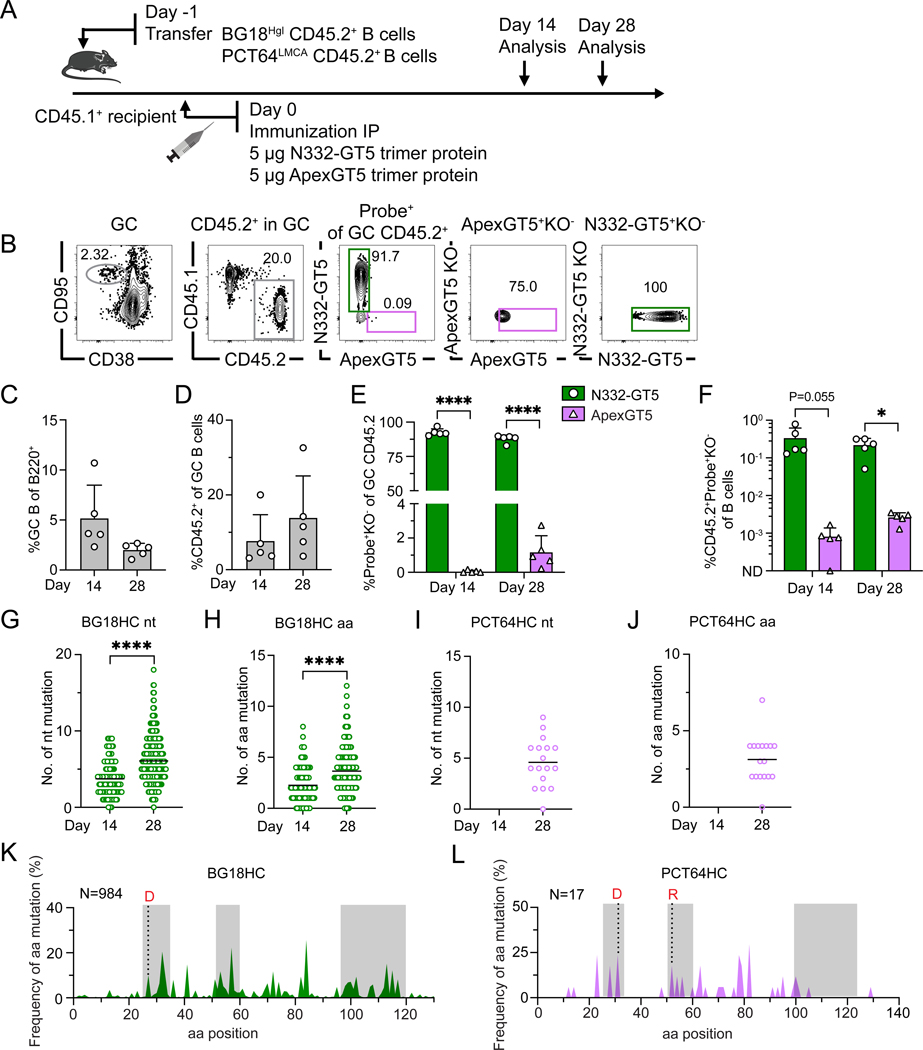
Limited co-activation of BG18-class/V3-glycan and PCT64-class/V2-apex bnAb precursors after dual delivery of BG505 SOSIP MD39-derived protein trimers. (**A**) Schematic showing naïve CD45.2^+^ BG18^Hgl^ (post-transfer frequency titrated to 7:10^6^) and PCT64^LMCA^ (post-transfer frequency = 10:10^6^) B cells adoptively transferred into CD45.1 WT mice (day −1); recipients were immunized with both N332-GT5 and ApexGT5 trimer protein via intraperitoneal (IP) injection. Samples were assayed 14 and 28 days post-immunization (dpi). Experiments were performed twice, and representative data from one are shown. (**B**) Gating strategy plots showing germinal center (GC), CD45.2 cells in GC, N332-GT5 and ApexGT5 binders of CD45.2 cells in GC and binding specificity after immunization. (**C–F**) Frequencies of GC B cells, CD45.2^+^ B cell in GC, V3-glycan epitope specific (N332-GT5^+^KO^−^) and Apex epitope-specific (ApexGT5^+^KO^−^) binders of GC CD45.2^+^ cells gated as in (B), and CD45.2 epitope-specific binders in total B cells 14 and 28 dpi; n=5 in both time points. Statistics generated using Welch’s t test in (E & F). Not detected (ND). Bars indicate mean + SD. *P < 0.05; ****P < 0.0001. (**G–J**) Heavy chain nucleotide (nt) and amino acid (aa) mutations across all sites of BCRs isolated from CD45.2^+^Probe^+^KO^−^IgD^−^ B cells 14 and 28 dpi: gl BG18 HC nt (G); gl BG18 HC aa. Statistics generated using Mann-Whitney test in (G & H). Bars indicate mean. ****P < 0.0001. (H); LMCA PCT64 HC nt (I); LMCA PCT64 HC aa (J). LMCA PCT64 sequences were not recovered on day 14. Bars indicate mean. (**K–L**) Per site heavy chain aa mutation frequency 28 dpi. CDRs boxed in grey; key mature (red) mutations were marked with letters; sequence number analyzed marked on the top left side. gl BG18 HC (K). LMCA PCT64 HC (L).

BnAbs to the V3-glycan/N332 high mannose patch epitope show encouraging neutralization (51–54); the most potent identified to date is BG18 (55). Previously, we established the mouse model BG18^Hgl^, where B cells express the BG18 inferred germline (iGL) heavy chain (HC) paired in BCRs with murine light chains (LCs) (14, 49). The immunogen N332-GT5 gp151 (N332-GT5 for brevity) (14), which is now in a Phase 1 clinical trial (HVTN144; NCT06033209) based on strong performance in murine and non-human primate (NHP) models (21, 24), binds naïve BG18^Hgl^ epitope-specific Fabs (mean K_D_ = 9.3 nM) and can elicit responses in BG18^Hgl^ as either a soluble protein trimer or a membrane-anchored protein encoded by mRNA-LNP (21). The V2-apex-specific bnAb PCT64 is also an attractive vaccine target with substantial HC precursors in humans and relatively low rates of SHM required to reach maturity (26, 51, 56–58). We previously developed a model bearing a least mutated common ancestor (LMCA)-reverted HC paired with either a human PCT64 germline LC or murine LCs, PCT64^LMCA^ (25). The trimer immunogen ApexGT5 gp151 (ApexGT5 for brevity) binds human PCT64^LMCA^ (K_D_ = 66 nM) (26) and can activate PCT64^LMCA^ precursors in PCT64^LMCA^ as a soluble protein trimer adjuvanted with Sigma or when encoded as a membrane-anchored protein by mRNA-LNP (25). Both N332-GT5 and ApexGT5 were designed around the BG505 SOSIP MD39 trimer (13), but with the addition of GT mutations to confer affinity for BG18-class precursors or PCT64- and PG9-class precursors, respectively (14, 26). To determine whether germline precursors to BG18 and PCT64 could be activated simultaneously using immunogens designed around the same stabilized trimer, we adoptively transferred B cells from both the BG18^Hgl^ and PCT64^LMCA^ CD45.2 knockin mouse lines into congenic CD45.1 WT mice, establishing low, stringent frequencies within the ranges previously used for immunogen testing in these models (21, 25). Co-transfer recipient mice were immunized intraperitoneally (IP) with both 5 μg of N332-GT5 and ApexGT5 soluble protein trimers formulated in Sigma adjuvant (Sigma, #S6322 SIGMA), and B cell responses were analyzed at days 14 and 28 post-prime (Fig. 1A). Protein co-administration elicited robust GC reactions on day 14 (5.2%), which were diminished but still present on day 28 (2.0%) (Fig. 1, B and C). GCs contained CD45.2^+^ precursor B cells at both time points (Fig. 1, B and D); however, this subpopulation consisted of N332-GT5 binders, rather than ApexGT5^+^ cells, almost exclusively (Fig. 1, B, E and F). We examined the immunogen batch and confirmed that ApexGT5 protein eluted primarily as a single peak, exhibited a molecular weight within the anticipated range, and demonstrated strong binding affinity for PCT64 LMCA (K_D_=40 nM) (fig. S1), suggesting that the weak PCT64^LMCA^ activation observed was not due to immunogen quality.

Most known HIV bnAbs undergo a high number of mutations from germline to achieve affinity maturation (6). Having established that BG18 and some PCT64 precursors entered GCs after co-administration, we investigated somatic hypermutation (SHM) in both lineages. We sorted CD45.2^+^ antigen (Ag)-specific B cell binders and performed 10x BCR sequencing after dual trimer protein immunization (fig. S2A). N332-GT5 and ApexGT5 WT probes were labelled with the same double fluoresceins; epitope knockout (KO) probes were labelled with a third fluorescein, after which we performed an unbiased probe-binding sort. In line with the flow cytometry results, 127 and 984 BG18 sequences were harvested at day 14 and 28, respectively; in contrast, only 17 PCT64 sequences were recovered at day 28 and none were recovered at day 14 (Fig. 1, G to J). The gl BG18 HCs accumulated both nucleotide (nt) and amino acid (aa) mutations over time (Fig. 1, G and H). Mutations of both gl BG18 and LMCA PCT64 HCs at day 28 were enriched in the HCDRs, which also displayed key mature (G27D in BG18; N31D and K52R in PCT64) mutations (Fig. 1, K and L). In sum, while BG18-class/V3-glycan and PCT64-class/V2-apex bnAb precursors were both activated by coadministration of N332-GT5 and ApexGT5 trimer protein, leading to SHM in both lineages, the ApexGT5 response in GCs was weak.

### Increased precursor frequencies enhance PCT64-class/V2-apex responses after dual protein trimer immunization

**Figure 2.**
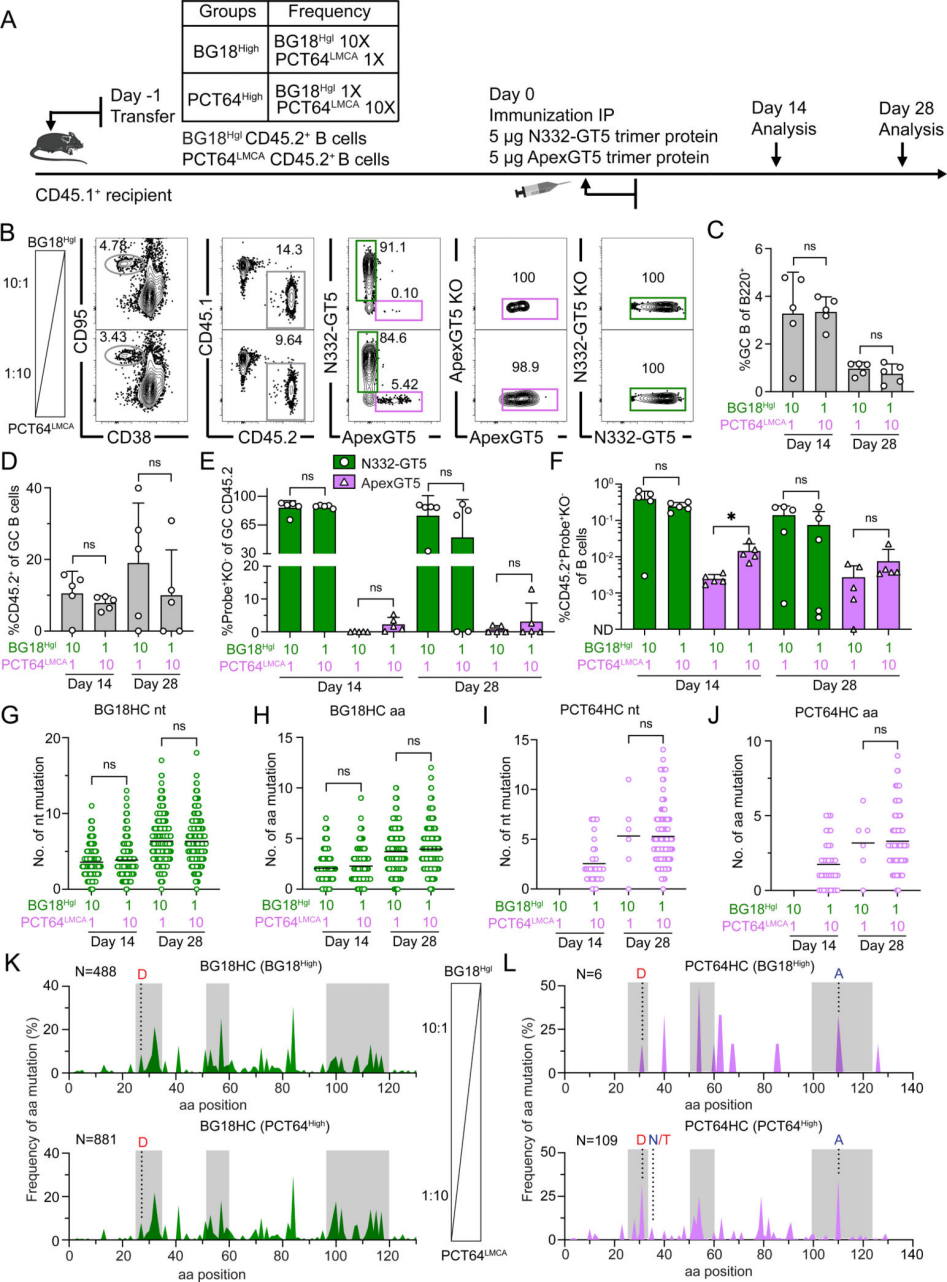
Increased precursor frequency improves dual activation of PCT64-class/V2-apex and BG18-class/V3-glycan bnAb precursors by protein trimers. (**A**) Schematic showing naïve CD45.2 BG18^Hgl^ and PCT64^LMCA^ B cells adoptively transferred to CD45.1 WT mice (day −1) to establish distinct starting precursor frequency ratios. “1/1X” post-transfer frequencies for BG18^Hgl^ and PCT64^LMCA^ were as in prior experiments (approximately 7:10^6^ and 10:10^6^, respectively) while “10/10X” indicates respective post-transfer frequencies of 70:10^6^ and 100:10^6^. Recipients were immunized IP with both N332-GT5 and ApexGT5 trimer protein (day 0). Samples were collected 14 and 28 dpi for analysis. Data was collected from a single run. (**B**) Gating strategy plots showing GC, CD45.2 cells in GC, N332-GT5 and ApexGT5 binders of CD45.2 cells in GC and binding specificity after immunization. (**C–F**) Frequencies of GC B cells, CD45.2^+^ B cell in GC, V3-glycan epitope specific (N332-GT5^+^KO^−^) and Apex epitope-specific (ApexGT5^+^KO^−^) binders of GC CD45.2^+^ cells gated as in (B), and CD45.2 epitope-specific binders in total B cells at 14 and 28 dpi; n=5 in each condition. Statistics generated using One-Way ANOVA analysis with Tukey’s multiple comparison test in (C), Brown-Forsythe and Welch ANOVA with Dunnett’s T3 multiple comparison (D), Welch’s or Unpaired t test in (E & F). Not detected (ND). Bars indicate mean + SD. Not significant (ns); *P < 0.05. (**G–J**) Heavy chain nucleotide (nt) and amino acid (aa) mutations across all sites of BCRs isolated from CD45.2^+^Probe^+^KO^−^IgD^−^ B cells 14 and 28 dpi. gl BG18 HC nt (G); gl BG18 HC aa (H); LMCA PCT64 HC nt (I); LMCA PCT64 HC aa (J). Statistics generated using Kruskal-Wallis test. Bars indicate mean. Not significant (ns). Note: LMCA PCT64 sequences in BG18^High^ frequency group were not recovered on day 14. (**K–L**) Per site heavy chain aa mutation frequency at 28 dpi. CDRs boxed in grey; key mature (red) and on-track (dark blue) mutations for PCT64 were marked with letters (25, 56); sequence number analyzed marked on the top left side. gl BG18 HC: BG18^High^ (top); PCT64^High^ (bottom) (K). LMCA PCT64 HC: BG18^High^ (top); PCT64^High^ (bottom) (L).

Precursor frequency can be a major determinant of the magnitude and quality of the response to immunogens (59). To investigate whether the limited PCT64-class/V2-apex response to protein could be rescued by altering precursor frequencies, we adoptively transferred CD45.2 BG18^Hgl^ and PCT64^LMCA^ B cells at variable starting frequencies into the same murine hosts. BG18^Hgl^ was titrated to a frequency of either 7:10^6^ (referred to as 1x) or 70:10^6^ (10x), PCT64^LMCA^ at 10:10^6^ (1x) or 100:10^6^ (10x), to produce models with either a high starting BG18^Hgl^ B cell frequency, BG18^High^ (BG18^Hgl^ 10x:PCT64^LMCA^ 1x), or a high PCT64^LMCA^ B cell frequency, PCT64^High^ (BG18^Hgl^ 1x:PCT64^LMCA^ 10x). We then primed the recipients with both N332-GT5 and ApexGT5 trimer protein in Sigma adjuvant and collected samples 14 and 28 dpi for analysis (Fig. 2A). Dual immunization induced similar GC sizes and CD45.2 cell participation in GCs at both frequencies (Fig. 2, B to D). The GC CD45.2 population primarily consisted of N332-GT5 binders no matter the initial precursor frequency; however, the PCT64^High^ condition did allow for increased ApexGT5^+^ CD45.2 B cell participation in GCs in several mice (Fig. 2, B and E) and a significantly higher proportion of CD45.2^+^ ApexGT5 binders in total B cells at day 14 (Fig. 2F).

To determine whether altered starting frequencies affected SHM for either gl BG18 or LMCA PCT64, CD45.2^+^ epitope-specific binders were sorted and single-cell BCR sequencing was performed as above. Both gl BG18 and LMCA PCT64 HCs accumulated mutations over time, although LMCA PCT64 sequences were not recovered at day 14 in the BG18^High^ treatment. Differential precursor frequency led to no significant differences in gl BG18 or LMCA PCT64 mutations, though the absence of LMCA PCT64 sequences in the BG18^High^ condition at day 14 and the low number at day 28 limited the conclusiveness of this observation (Fig. 2, G to J). Gl BG18 HC hotspot mutation patterns were almost identical regardless of initial precursor frequency (Fig. 2K). LMCA PCT64 HC mutation patterns were more variable, likely due to limited sequence recovery in the BG18^High^ condition, but they exhibited similar key mature mutations as well as intermediate “on-track” mutations identified from the PCT64 lineage (Fig. 2L) (25, 56). In sum, increased starting frequencies improved the participation of PCT64^LMCA^ B cells in the GC after dual protein immunization and allowed both lineages to undergo SHM.

### An mRNA-LNP cocktail activates both BG18-class/V3-glycan and PCT64-class/V2-apex bnAb precursors efficiently

**Figure 3.**
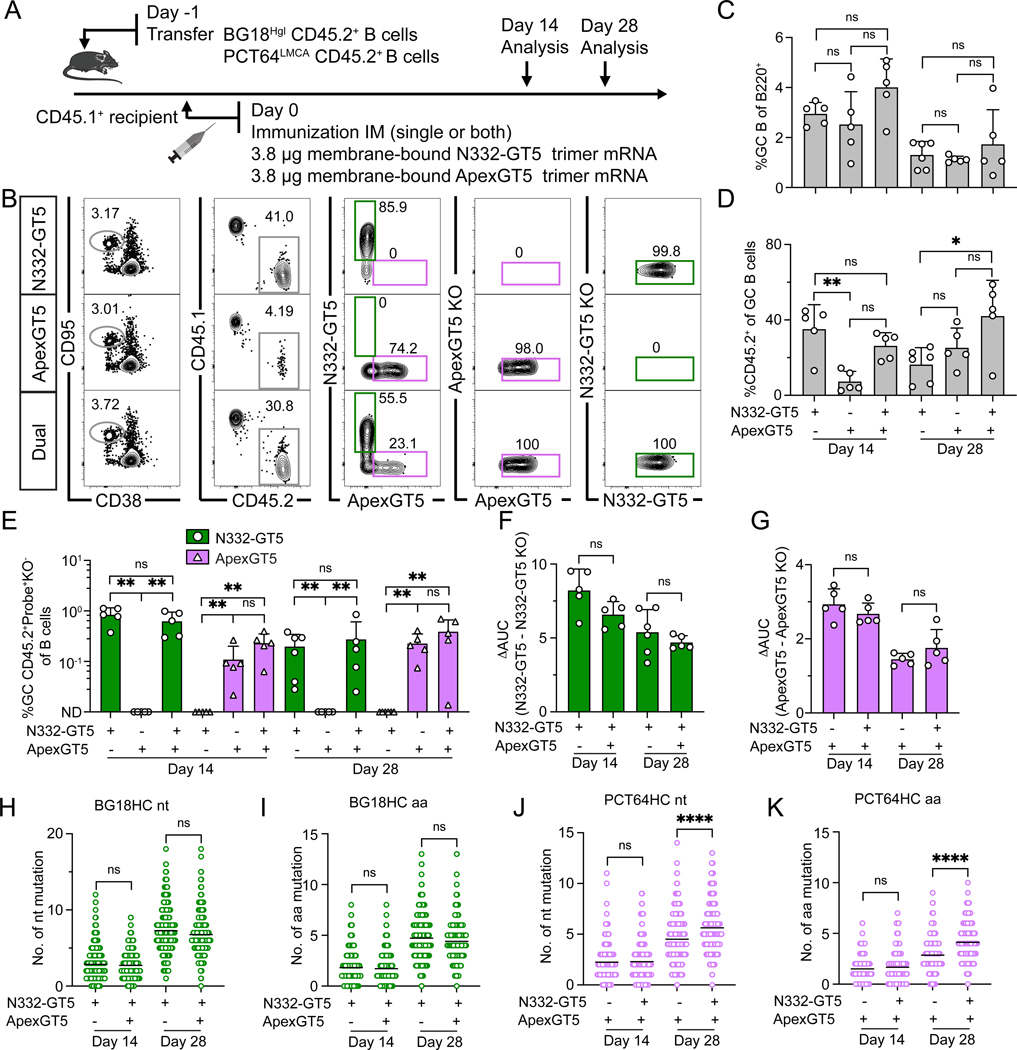
An mRNA-LNP cocktail efficiently activates both PCT64-class/V2-apex and BG18-class/ V3-glycan bnAb precursors. (**A**) Schematic showing naïve CD45.2 BG18^Hgl^ (post-transfer frequency = 7:10^6^) and PCT64^LMCA^ (post-transfer frequency = 10:10^6^) B cells adoptively transferred into CD45.1 WT recipients (day −1), which were then immunized with either single N332-GT5, ApexGT5 trimer Mrna-LNP, or both via intramuscular (IM) injection (day 0). Samples were collected at 14 and 28 dpi. Experiments were performed twice, and representative data from one experiment are shown. (**B**) Gating strategy plots showing GC, CD45.2 cells in GC, N332-GT5- and ApexGT5-binders among GC CD45.2 cells and binding specificity after immunization. (**C–E**) Frequencies of GC B cells (C), CD45.2^+^ B cells in GC (D), V3-glycan-specific (N332-GT5^+^KO^−^) and Apex-specific (ApexGT5^+^KO^−^) GC CD45.2^+^ cells in total B cells in each condition at 14 and 28 dpi (E). Each symbol represents a different mouse, n=5–6 in each condition. Statistics generated using Brown-Forsythe and Welch ANOVA with Dunnett’s T3 multiple comparison (C), One-Way ANOVA analysis with Tukey’s multiple comparison test in (D), Mann-Whitney or Unpaired t test in (E). Not detected (ND). Bars indicate mean + SD. Not significant (ns); *P < 0.05; **P < 0.01. (**F–G**) Serum IgG antibody titer measurement. ΔAUC (delta area under the curve) values were compared for N332-GT5 and N332-GT5 KO titers (F) or ApexGT5 and ApexGT5 KO titers (G). Each symbol represents a different mouse with 5–6 mice at each time point. Bars represent mean + SD. Statistical analysis was performed using One-Way ANOVA with Tukey’s multiple comparison test. Bars indicate mean + SD. Not significant (ns). (**H–K**) Number of heavy chain nt and aa mutations across all sites of BCRs isolated from CD45.2^+^Probe^+^KO^−^IgD^−^ B cells at 14 and 28 dpi. gl BG18 HC nt (H); gl BG18 HC aa (I); LMCA PCT64 HC nt (J); LMCA PCT64 HC aa (K). Statistical analysis was performed using Kruskal-Wallis test. Bars indicate mean. Not significant (ns); ****P < 0.0001.

While the number of potential precursors can be increased by GT mutations during the design of the priming immunogen (14), it is not feasible to increase the starting precursor frequency to enhance responses to an existing immunogen in humans. We previously demonstrated that the membrane-anchored ApexGT5 encoded by mRNA-LNP lowers the affinity threshold required to activate PCT64 precursors relative to soluble ApexGT5 trimer protein (25). To determine whether this held true in a dual vaccination regimen, CD45.1 WT recipients were co-transferred with CD45.2 BG18^Hgl^ and PCT64^LMCA^ cells and then immunized intramuscularly (IM) with mRNA-LNPs encoding membrane-anchored trimers of either N332-GT5, ApexGT5, or both at a dose of 3.8 μg each and assayed 14 and 28 dpi (Fig. 3A). There was no significant difference in GC sizes between single or dual immunization at day 14 or 28 (Fig. 3, B and C). N332-GT5 alone recruited more CD45.2 cells to GCs than ApexGT5 alone at day 14, but the two were indistinguishable at day 28; CD45.2 as a fraction of GCs at day 28 was highest after dual administration (~40%), but this was significant only in comparison to N332-GT5 alone (Fig. 3D). As a fraction of total B cells, GC CD45.2^+^ N332-GT5^+^KO^−^ and ApexGT5^+^KO^−^ were not negatively affected by dual relative to single delivery of their respective immunogens; ApexGT5^+^KO^−^ cells seemed to be somewhat enhanced, but this was not significant (Fig. 3E). Serum IgG levels against N332-GT5 and ApexGT5 were also similar after single and dual immunizations (Fig. 3, F and G, and fig. S3, A and B). gl BG18 HCs acquired nt and aa mutations at the same frequency after single or dual immunization (Fig. 3, H and I). LMCA PCT64 HCs, however, displayed more mutations at day 28 after dual immunization than after ApexGT5 alone (Fig. 3, J and K). Hotspot mutations in both gl BG18 and LMCA PCT64 HCs developed in similar HCDR locations between dual immunization and their respective single immunizations, though the mutation rate at the G110 site in LMCA PCT64 was higher after dual than single ApexGT5 immunization (fig. S3, C and D).

To determine whether the improved Apex response might be driven by the membrane-bound presentation of mRNA-LNP ApexGT5 or, alternatively, other effects of mRNA-LNP formulation, we transferred PCT64^LMCA^ cells and subsequently primed the mice with mRNA-LNP encoding ApexGT6 (27), a related ApexGT priming immunogen, in either a membrane-bound or soluble format (fig. S4A). The membrane-bound mRNA induced larger GC sizes 10 dpi (fig. S4, B and C) and recruited more CD45.2^+^ cells for activation and expansion (fig. S4, B and D to F).

In sum, a cocktail of N332-GT5 and ApexGT5 membrane-bound trimer mRNA-LNPs improved the activation of PCT64 LMCA precursors relative to soluble protein co-administration and allowed them to undergo SHM. Membrane-bound antigen presentation may underly this difference by lowering the precursor activation threshold.

### BG18-class/V3-glycan and PCT64-class/V2-apex respond to mRNA-LNP cocktails when potential competitors are abundant

**Figure 4.**
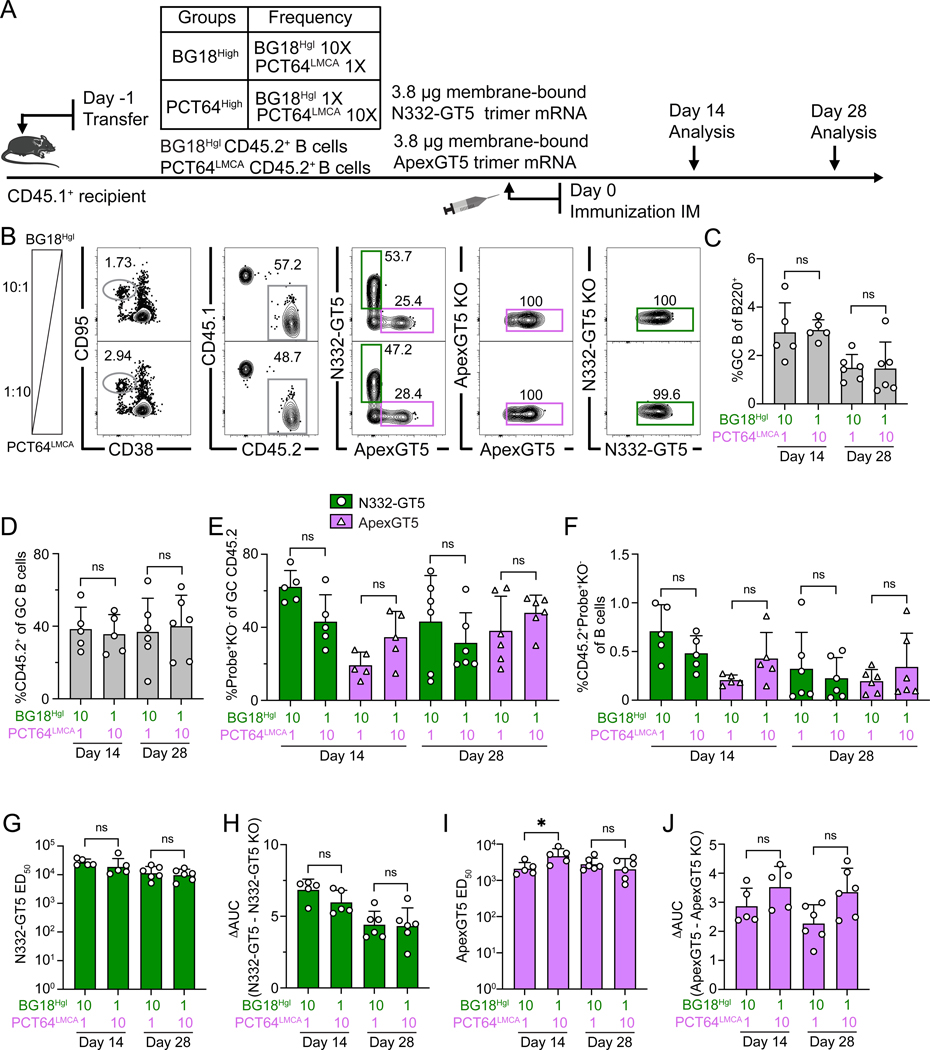
Simultaneous activation of BG18-class/V3-glycan and PCT64-class/V2-apex-responses by mRNA-LNP immunization at variable precursor frequencies. (**A**) Schematic showing naïve CD45.2 BG18^Hgl^ and PCT64^LMCA^ B cells adoptively transferred into CD45.1 WT mice to establish distinct starting frequencies (day −1). “1/1X” post-transfer frequencies for BG18^Hgl^ and PCT64^LMCA^ were as in prior experiments (approximately 7:10^6^ and 10:10^6^, respectively) while “10/10X” indicates respective post-transfer frequencies of 70:10^6^ and 100:10^6^. Recipients were then immunized with both N332-GT5 and ApexGT5 trimer mRNA-LNPs via IM injection (day 0). Samples were collected 14 and 28 dpi. Experiments were performed twice, and representative data from one experiment are shown. (**B**) Gating strategy plots showing GC, CD45.2 cells in GC, N332-GT5 and ApexGT5 binders among GC CD45.2 cells, and binding specificity after immunization. (**C–F**) Frequencies of GC B cells (C), CD45.2^+^ B cell in GC (D), V3-glycan epitope specific (N332-GT5^+^KO^−^) and Apex epitope specific (ApexGT5^+^KO^−^) binders among GC CD45.2^+^ cells (E), and CD45.2 epitope-specific binders in total B cells (F) 14 and 28 dpi; n=5–6 in each condition. Statistics generated using One-Way ANOVA with Tukey’s multiple comparison test in (C–E), Brown-Forsythe and Welch’s ANOVA with Dunnett’s T3 multiple comparison (F) and. Bars indicate mean + SD. Not significant (ns). (**G–J**) Serum antibody titer measurements. Serum IgG 50% equilibrium dilution (ED_50_) values for N332-GT5 (G) or ApexGT5 (I) at 14 and 28 dpi. ΔAUC comparison for N332-GT5 and N332-GT5 KO titer (H), ApexGT5 and ApexGT5 KO titer (J). Each symbol represents a different mouse with 5–6 mice in each condition. Bars represent geometric mean + geometric SD (G & I) or indicate mean + SD (H & J). Statistics generated using Brown-Forsythe and Welch’s ANOVA with Dunnett’s T3 multiple comparison (G) and One-Way ANOVA with Tukey’s multiple comparison test in (H–J). Not significant (ns); *P < 0.05.

To determine whether the responses to the N332-GT5 and ApexGT5 mRNA-LNP cocktail were similarly affected by precursor frequency, we again established BG18^High^ and PCT64^High^ models. We primed the recipients with both N332-GT5 and ApexGT5 mRNA-LNPs and collected samples 14 and 28 dpi for analysis (Fig. 4A). Dual immunization induced similar GC sizes at both frequencies (Fig. 4, B and C), and CD45.2 B cell occupation of GCs was similar (~40%) in both cohorts at days 14 and 28 (Fig. 4D). Higher starting frequencies generally increased Probe^+^KO^−^ B cells targeting corresponding epitopes as a fraction of either GC CD45.2 B cells or total B cells, but these increases were not significant (Fig. 4, E and F). Low BG18^Hgl^ starting frequency had no effect on the serum IgG levels of N332-GT5 antibodies (Fig. 4, G and H), and, though ApexGT5 ED_50_ was lower at day 14 in the low PCT64^LMCA^ transfer model, this gap equilibrated by day 28, and epitope-specific ApexGT5 antibodies were indistinguishable at both time points (Fig. 4, I and J). Changing the starting frequencies within a 10-fold range therefore had minimal impact on the response.

To determine whether starting frequency alterations affected SHM, CD45.2^+^ epitope-specific binders were sorted and single-cell BCR sequencing was performed as above. Both gl BG18 and LMCA PCT64 HCs accumulated mutations over time, and there were no significant differences in total nt or aa mutations of gl BG18 related to starting frequency; although slightly more aa (day 14) and nt (day 28) mutations were observed in LMCA PCT64 HCs when PCT64^LMCA^ starting frequencies were higher (fig. S5, A to D). Mutation patterns were also convergent regardless of initial precursor frequency (fig. S5, E and F). Thus, while a 10-fold change in precursor frequency could improve V2-Apex responses to protein immunization, the mRNA-LNP format were not sensitive to precursor frequency changes within this range.

### VRC01-class bnAb precursors to the CD4bs can be activated in tandem with PCT64-class/V2-apex or BG18-class/V3-glycan bnAb precursors

**Figure 5.**
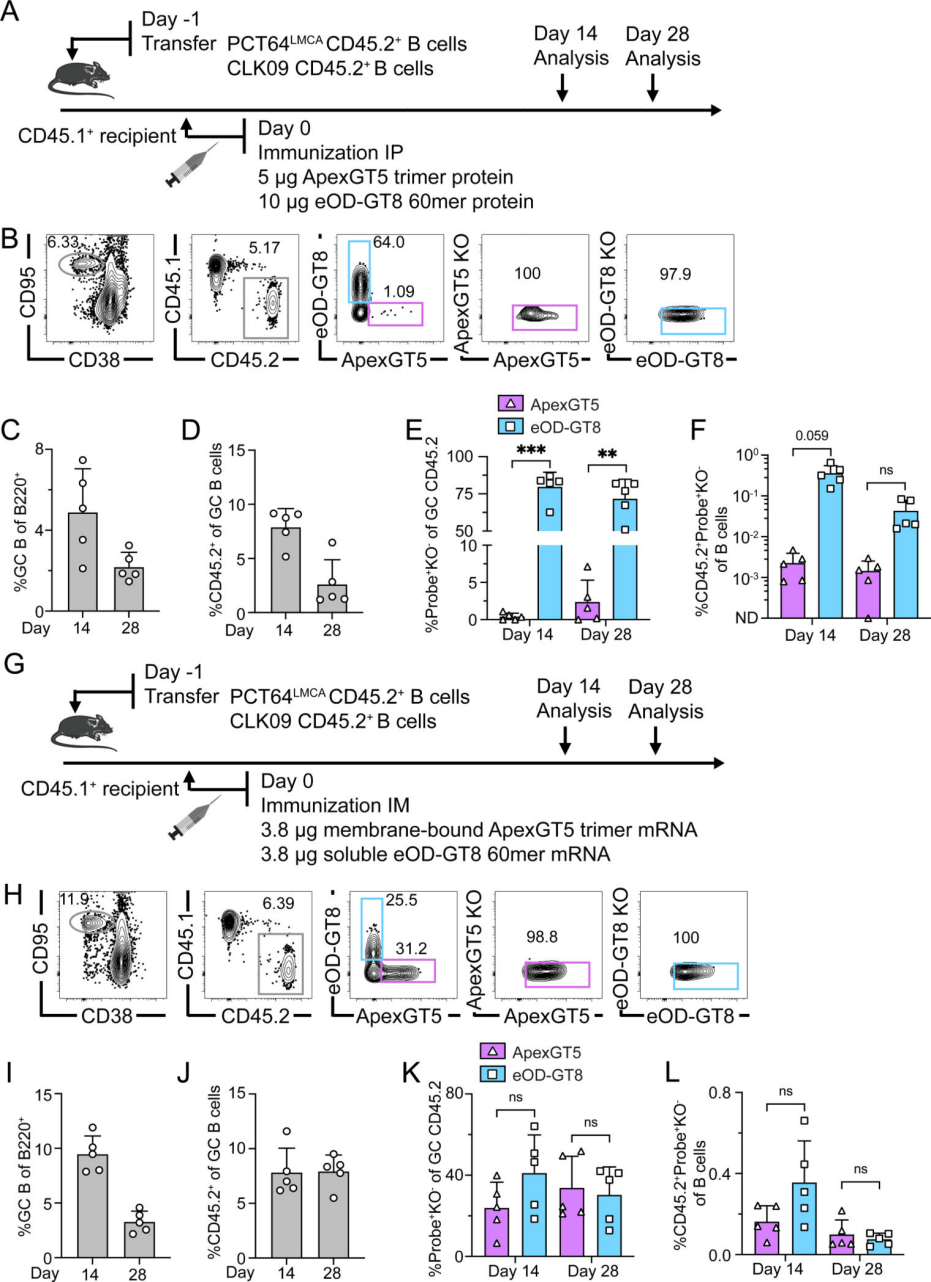
PCT64-class/V2-apex and VRC01-class/CD4bs bnAb precursors can be activated simultaneously with a trimer and nanoparticle immunogen. (**A**) Schematic showing naïve CD45.2 PCT64^LMCA^ (post-transfer frequency = 10:10^6^) and CLK09 (post-transfer frequency = 20:10^6^) B cells adoptively transferred into CD45.1 WT mice (day −1); recipients were immunized with both ApexGT5 trimer and eOD-GT8 60mer protein via IP injection. Samples were assayed at 14 and 28 dpi. Data collected from a single run. (**B**) Gating strategy plots showing GC, CD45.2 cells in GC, ApexGT5 and eOD-GT8 binders of CD45.2 cells in GC, and epitope binding specificity after protein coadministration. (**C–F**) Frequencies of GC B cells (C), CD45.2^+^ B cell in GC (D), Apex epitope-specific (ApexGT5^+^KO^−^) and CD4bs-epitope-specific (eOD-GT8^+^KO^−^) binders of GC CD45.2^+^ cells (E) gated as in (B) and CD45.2^+^ epitope specific binders in total B cells (F) at 14 and 28 dpi; n=5 in both time points. Statistics generated using Brown-Forsythe and Welch’s ANOVA with Dunnett’s T3 multiple comparison in (E, F). Not detected (ND). Bars indicate mean + SD. Not significant (ns); **P < 0.01; ***P < 0.001. (**G**) Schematic showing naïve CD45.2 PCT64^LMCA^ (post-transfer frequency = 10:10^6^) and CLK09 (post-transfer frequency = 20:10^6^) B cell adoptive transfer recipients immunized with both ApexGT5 trimer and eOD-GT8 60mer mRNA-LNPs via IM injection. Samples were collected at 14 and 28 dpi. Data collected from a single run. (**H**) Gating strategy plots showing GC, CD45.2 cells in GC, ApexGT5 and eOD-GT8 binders of CD45.2 cells in GC and epitope binding specificity after administration of mRNA-LNP cocktail. (**I–L**) Frequencies of GC B cells (I), CD45.2^+^ B cell in GC (J), Apex epitope specific (ApexGT5^+^KO^−^) and CD4bs epitope specific (eOD-GT8^+^KO^−^) binders of GC CD45.2^+^ cells (K) gated as in (H) and CD45.2^+^ epitope specific binders in total B cells (L) 14 and 28 dpi; n=5 in both time points. Statistics generated using One-Way ANOVA with Tukey’s multiple comparison test in (K) and Brown-Forsythe and Welch’s ANOVA with Dunnett’s T3 multiple comparison (L). Bars indicate mean + SD. Not significant (ns).

A number of GT immunogens are designed around VRC01-class bnAbs targeting the CD4bs (9, 12, 17, 22, 23, 60), including an engineered outer domain of HIV gp120 (eOD-GT8) presented as 60 copies on a self-assembling soluble nanoparticle (60mer NP) (23, 61). Previously, we established the CLK family of mouse models (50), wherein B cells express germline knockin HC and LCs of VRC01 bnAb precursors (62) which are efficiently activated by the eOD-GT8 60mer NP (50). To determine whether VRC01-class precursors could participate in multi-site activation responses after the co-administration of protein trimer and NP immunogens, we adoptively transferred precursor B cells from PCT64^LMCA^ and CLK09 (which binds eOD-GT8 at K_D_ = 350 nM (62)) CD45.2 knockin mouse lines into congenic CD45.1 WT mice (25, 50) at physiologically relevant frequencies previously used for this immunogen and model combination (20, 25) and immunized recipients with both 5 μg of ApexGT5 soluble protein trimer and 10 μg eOD-GT8 soluble 60mer with Sigma adjuvants. B cell responses were analyzed at days 14 and 28 post-prime (Fig. 5A). Co-immunization with the protein immunogens induced robust GC responses with CD45.2 cells at both time points (Fig. 5, B to D), the vast majority of which were probe-positive for eOD-GT8 rather than ApexGT5 (Fig. 5, E and F). PCT64 and CLK09 HCs accumulated both nt and aa mutations over time, but, as expected from the flow cytometry results, while 539 and 1104 CLK09 sequences were isolated at day 14 and 28, respectively, only 6 and 42 PCT64 sequences were recovered on those time points (fig. S6, A to D). Mutations at day 28 were enriched in the HCDRs, which also displayed on-track (S35N, K52E, and G110A in PCT64) or key mature (N31D, S35T, and G110D in PCT64; G31D/A in CLK09) mutations (fig. S6, E and F).

eOD-GT8 60mer is also highly immunogenic when delivered by mRNA-LNP as a self-assembling soluble NP (19, 20). To determine whether ApexGT5 mRNA-LNP could again improve PCT64-class/V2-Apex precursor activation as part of an mRNA-LNP cocktail, we established a dual transfer model as above and immunized the recipients with a membrane-anchored ApexGT5 trimer and a soluble eOD-GT8 60mer mRNA-LNP (dose: 3.8 μg each) and assayed responses at the same time points (Fig. 5G). Similar to protein immunogens, co-delivery of both mRNA-LNPs elicited strong GC reactions on day 14, which remained, though diminished, until day 28 (Fig. 5, H and I). The GCs contained ~8% CD45.2 B cells at both time points (Fig. 5, H and J). In contrast to protein immunization, the GC CD45.2^+^ bnAb precursor repertoire consisted of 24.0% ApexGT5- and 41.1% eOD-GT8- specific binders on day 14, equalizing to ~30% on day 28 (Fig. 5, H and K). The proportion of both precursors was similar as a percentage of total B cells at day 28 (Fig. 5L). Co-immunization elicited serum IgG titer against both epitopes (fig. S6, G and I), and ΔAUC specific to each epitope were high at day 14 but waned at day 28 (fig. S6, H and J). HCs from both PCT64 and CLK09 gained SHM over time after dual mRNA-LNP (fig. S6, K to N), exhibiting similar mutation patterns and on-track or key mature mutations (fig. S6, E and F, O and P). Thus, while PCT64-class/V2-apex and VRC01-class/CD4bs bnAb precursors were activated simultaneously and underwent SHM after co-administration of ApexGT5 trimer and eOD-GT8 60mer, the ApexGT5 response in GCs was limited unless the immunogens were delivered as mRNA-LNPs.

To determine whether mRNA-LNP eOD-GT8 60mer could also drive efficient activation with the N332-GT5 mRNA-LNP trimer immunogen, we adoptively transferred BG18^Hgl^ and CLK09 precursors into a single host and immunized recipients with mRNA-LNPs encoding either N332-GT5 trimer, eOD-GT8 60mer or both at a dose of 10 μg each and assayed 14 and 28 dpi (fig. S7A). mRNA-LNP eOD-GT8 60mer on its own produced larger GCs than the N332-GT5 trimer mRNA-LNP, but co-delivery produced the largest GCs at both time points (fig. S7, B and C). The proportion of CD45.2 cells recruited to GCs after single N332-GT5 trimer immunization was higher than after eOD-GT8 60mer or dual delivery (fig. S7D). GC CD45.2^+^N332-GT5^+^KO^−^ were, as a fraction of total B cells, statistically indistinguishable between single N332-GT5 and dual immunization; while the same was not true for CD45.2^+^ eOD-GT8^+^ KO^−^ B cells at day 14, their numbers recovered by day 28 (fig. S7E). Overall serum titers and eOD-GT8 CD4bs epitope-specific IgG levels were similar across treatments (fig. S7, F, H and I), though serum N332-GT5 V3-glycan epitope-specific IgG levels did vary between single N332-GT5 immunization and dual immunization, but in opposing directions at days 14 and 28 (fig. S7G). In terms of total SHM, dual immunization was either neutral or beneficial (fig. S8, A to D), and the same key mature mutations and similar hotspot mutation patterns emerged in BG18 and CLK09 HCs (fig. S8, E and F). In sum, precursors to two distinct bnAb classes could be activated and undergo SHM after immunization by mRNA-LNP cocktails containing eOD-GT8 60mer and either trimer.

### Germline precursors to bnAbs targeting three distinct epitopes can be activated simultaneously

**Figure 6.**
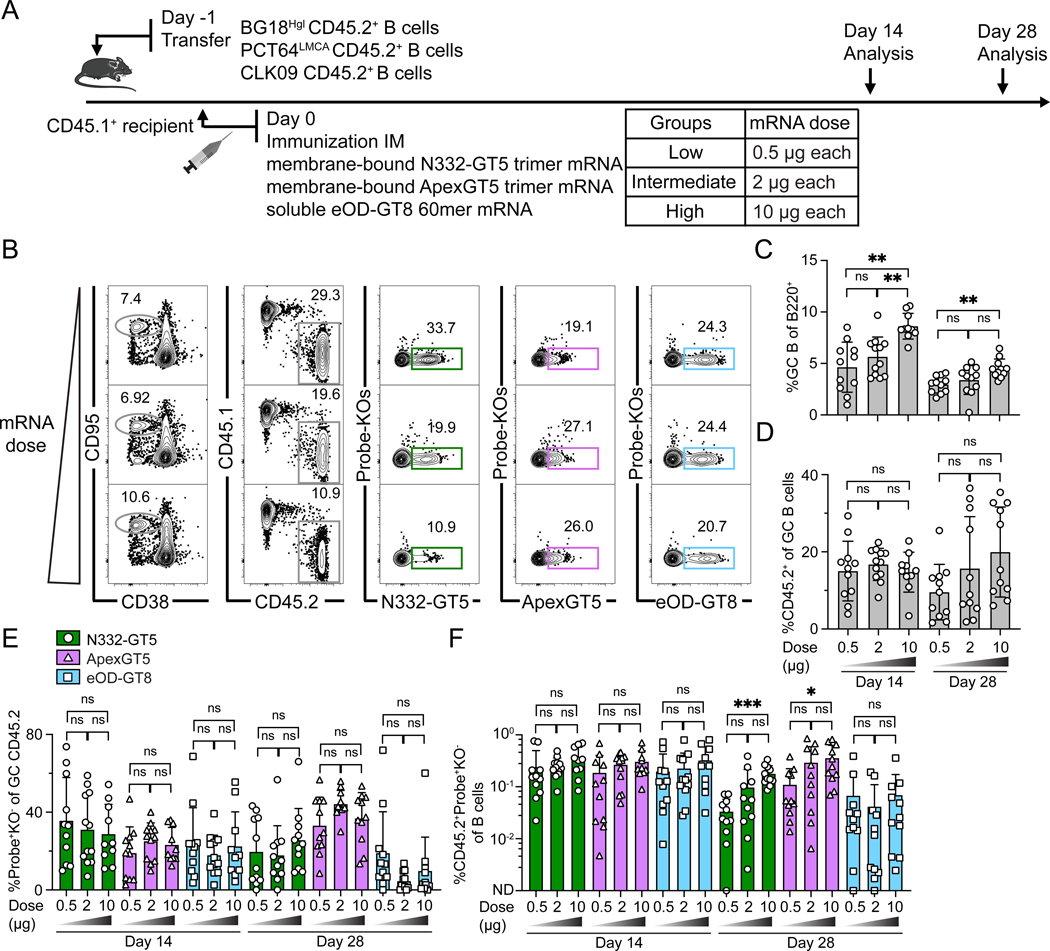
BG18-class/V3-glycan, PCT64-class/V2-apex, and VRC01-class/CD4bs bnAb precursor responses can be elicited simultaneously. (**A**) Schematic showing naïve CD45.2 BG18^Hgl^ (post-transfer frequency = 7:10^6^), PCT64^LMCA^ (post-transfer frequency = 10:10^6^), and CLK09 (post-transfer frequency = 20:10^6^) B cell adoptive transfer recipients immunized with triple mRNA-LNP immunogens (N332-GT5 trimer, ApexGT5 trimer, and eOD-GT8 60mer) simultaneously via IM injection. Samples were collected at 14 and 28 dpi. Data were pooled and shown from two experiments (n=10–12). (**B**) Gating strategy plots showing GC, CD45.2 cells in GC, N332-GT5, ApexGT5 and eOD-GT8 epitope-specific binders among GC CD45.2 cells after immunization. (**C–F**) Frequencies of GC B cells (C), CD45.2^+^ B cells in GC (D), V3-glycan epitope specific (N332-GT5^+^KO^−^), Apex-specific (ApexGT5^+^KO^−^) and CD4bs-specific (eOD-GT8^+^KO^−^) binders of GC CD45.2^+^ cells (E) gated as in (B), and CD45.2 epitope specific binders in total B cells (F) at 14 and 28 dpi; n=10–12 in each condition. Statistics generated using Brown-Forsythe and Welch’s ANOVA with Dunnett’s T3 multiple comparison (C & E), One-Way ANOVA with Tukey’s multiple comparison and t test (D & F). Not detected (ND). Bars indicate mean + SD. Not significant (ns); *P < 0.05; **P < 0.01; ***P < 0.001.

Having interrogated two-way interactions between precursor classes, we advanced to triple activation. We first examined triple activation in CD45.1 WT mice adoptively transferred with each precursor after protein immunization and found that, as after dual immunization with protein, ApexGT5 responses were present but weak (fig. S9, A to F). BG18 and CLK09 precursor HCs accumulated nt/aa mutations over time (fig. S9, G and H, K and L); however, only two LMCA PCT64 sequences were recovered (fig. S9, I and J). We therefore moved to immunization with a cocktail of all three mRNA-LNP immunogens (N332-GT5 trimer, ApexGT5 trimer and eOD-GT8 60mer) IM at a low dose (0.5 μg of each immunogen), an intermediate dose (2 μg each), or a high dose (10 μg each); responses were assayed at 14 and 28 dpi (Fig. 6A). Higher doses generally increased total GC size (Fig. 6, B and C), but total CD45.2 B cells in GCs were not affected by dose on average and displayed high variability (Fig. 6D). Binders to all three probes were found in CD45.2 GC B cells (Fig. 6E) and total CD45.2 B cells (Fig. 6F), with significant differences appearing only in total B cells and on day 28 for N332-GT5 and ApexGT5 binders, with a substantial variability across individual mice (Fig. 6F). Endogenous CD45.1 B cell responses in the mixed group were primarily against eOD-GT8 and did not vary significantly with dose (fig. S10, A to C).

**Figure 7.**
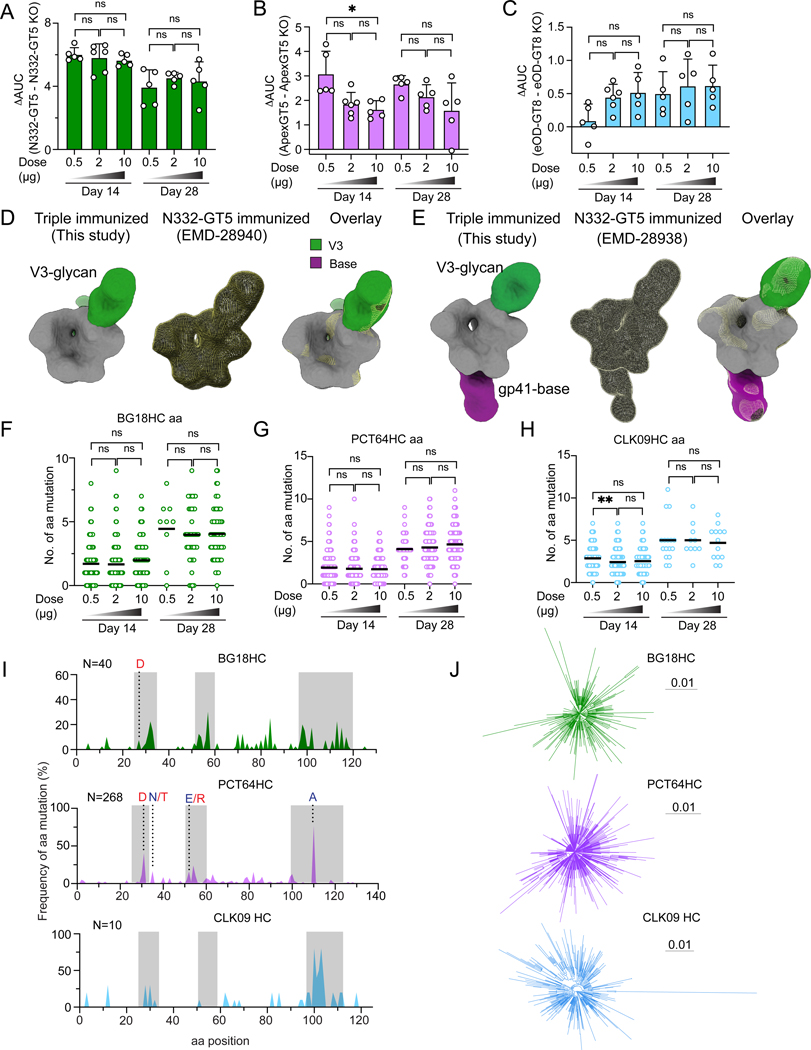
BG18^Hgl^, PCT64^LMCA^, and CLK09 produce serum antibody and undergo SHM during simultaneous activation. (**A–C**) ELISA quantification of N332-GT5-specific, ApexGT5-specific and eOD-GT8-specific IgG at 14 and 28 dpi. ΔAUC comparison of titers for N332-GT5 and N332-GT5-KO (A); ApexGT5 and ApexGT5-KO (B); eOD-GT8 and eOD-GT8-KO (C). Each symbol represents a different mouse with 5–6 mice in each condition. Statistics generated using One-Way ANOVA with Tukey’s multiple comparison test. Bars indicate mean + SD. Not significant (ns); *P < 0.05. (**D-E**) Electron microscopy polyclonal epitope mapping (EMPEM) following multi-priming immunization. A cocktail of N332-GT5 and ApexGT5 was used to form complexes with pooled serum IgG (digested to Fab) from triple mRNA-LNPs (high dose, panel D) or proteins (5μg N332-GT5, 5μg ApexGT5, 10μg eOD-GT8, panel E) primed mice at day 28. Responses against the V3-glycan epitope were detected in both study groups (green segment). An additional response against the gp41-base was detected in the protein group (purple segment). When compared to published EMPEM maps following priming with a single immunogen (N332-GT5), the angles of approach and binding positions of the V3-glycan responses are highly overlapping, even after simultaneous priming against additional epitopes. (**F–H**) Mutations across all sites of BCR heavy chains sequenced from CD45.2^+^Probe^+^KO^−^IgD^−^ B cells 14 and 28 dpi. gl BG18 HC aa (F); LMCA PCT64 HC aa (G); CLK09 HC aa (H). Statistical analysis performed using Kruskal-Wallis test. Bars indicate mean. Not significant (ns); **P < 0.01. (**I**) Per site heavy chain aa mutation frequency from intermediate dose 28 dpi. CDRs boxed in grey; key mature (red) or on-track (dark blue; PCT64-only) mutations marked with letters. N at top left indicates number of sequences included in analysis. gl BG18 HC (top green); LMCA PCT64 HC (middle pink); CLK09 HC (bottom blue). (**J**) Phylogenetic tree plots of each lineage after triple GT mRNA-LNP of intermediate dose 14 dpi. Each tree denotes a clonal lineage. Branch lengths are scaled by estimated heavy chain nt mutations. gl BG18 HC (top green); LMCA PCT64 HC (middle pink); CLK09 HC (bottom blue). Tree Scale (0.01) indicates the number of substitutions per site.

In terms of antibody production, total WT-antigen and epitope-KO-antigen IgG AUC generally increased with dose (fig. S11, A to F), but ΔAUC was similar across doses (Fig. 7, A to C). While otherwise dose-independent, epitope-specific CD45.2 plasmablast generation on day 28 did significantly increase for N332-GT5 and ApexGT5 at higher doses; CD45.1 epitope-specific plasmablasts either did not vary or, on day 28 for ApexGT5, decreased (fig. S10, D and E). Electron microscopy polyclonal epitope mapping (EMPEM) was used to assess whether serum antibodies against the V3-glycan and V2-apex epitopes were detectable after priming with the mRNA-LNP cocktail or protein cocktail. Polyclonal Fabs isolated from the sera of each study group were incubated with a cocktail of N332-GT5, ApexGT5, and eOD-GT8. As the mass of eOD-GT8 is too low for EMPEM, and the NP version too flexible for accurate alignment of particles by EM, it was excluded from downstream EMPEM analysis. EM 3D reconstructions revealed responses against the V3-glycan epitope in both study groups (Fig. 7, D and E), and additional responses to the gp41-base in the protein-only group. These EM maps have high overlap with published EMPEM maps obtained from single immunogen (N332-GT5) priming (21), confirming that the desired V3-glycan response can be elicited even in the presence of competing immunogens. V2-apex polyclonal antibodies were not detected, which correlated with the lower titers compared to V3-glycan antibodies and/or lower affinities of such antibodies at the day 28 time point.

To distinguish the serum IgG contributions from WT CD45.1 and transferred CD45.2 B cells, ELISA analysis was performed from samples of untransferred, WT CD45.1 mice and CD45.2 adoptive transfer recipients primed with an intermediate dose of triple mRNA-LNPs. Higher IgG ΔAUC to N332-GT5 antigen was in CD45.2 recipients compared with untransferred WT mice; the same outcome was observed for ApexGT5 and eOD-GT8 specific-epitopes, although only at day 14 (fig. S11, G to I). The IgG AUC to WT antigens varied with the epitope and time (fig. S11, J to L); however, the overall IgG AUC to epitope-KO antigens was lower in CD45.2-transferred relative to WT recipients (fig. S11, M to O), suggesting less off-target antibody production.

To determine whether SHM accumulated after triple mRNA delivery and whether mRNA dose affected maturation, CD45.2 epitope-specific B cell binders were collected and paired BCR sequences obtained via 10x Genomics. All precursor classes accumulated HC mutations over time at frequencies similar to those observed in dual activations, and immunogen dose did not affect mean nt and aa mutations for BG18 and PCT64 (Fig. 7, F to H and fig. S12, A to C); CLK09 showed fewer mutations at 14 days for the intermediate dose relative to the low dose, but numbers reached equilibrium by day 28 (Fig. 7H and fig. S12C). Mutational hotspots for BG18 and PCT64 HCs were also similar across doses (Fig. 7I and fig. S12, D and E). CLK09 HC hotspot mutation analysis found high mutation rates in CDR3, and variation in CDR1 and 2 mutation patterns across doses, though this was likely due to low sequence recovery at day 28 (Fig. 7I and fig. S12F). Phylogenetic analysis demonstrated that the heavy chains of each lineage diversified extensively post-prime (Fig. 7J and fig. S12, G to I). We then evaluated the neutralization ability of the IgGs purified from mouse serum after triple mRNA-LNP prime. No neutralization activity (IC50 >50 μg/ml) against native or modified HIV pseudoviruses sensitive to mature BG18, PCT64 or VRC01 bnAb was detected. However, high dose day 28 serum showed some activity against BG505_V1_GT5_ (fig. S13). In sum, BG18-class/V3-glycan, PCT64-class/V2-apex, and VRC01-class/CD4bs-directed bnAb precursors can be activated simultaneously and undergo affinity maturation across a range of mRNA-LNP doses, diversifying extensively. Nonetheless, they require further SHM to neutralize native pseudovirus potently and broadly.

### Precursors targeting four distinct epitopes can be activated simultaneously

HIV bnAbs to the MPER, such as 10E8, exhibit particularly high neutralization breadth (63). The mouse model HuGL18^H^ harbors the genuine human BCR HC of MPER-HuGL18 in association with endogenous murine LC; these BCRs can be activated by a 10E8-GT10.2 and GT10.3 12mer protein (for which MPER-HuGL18^H^ BCRs had median affinities of ~80 nM) even at initial precursor frequencies (100:10^6^) approximately 11-fold lower than the naïve human repertoire (28, 29). To examine whether priming could further expand to encompass precursors to this epitope, we adoptively transferred CD45.2 BG18^Hgl^ (7:10^6^), PCT64^LMCA^ (10:10^6^), CLK09 (20:10^6^) and HuGL18^H^ (100:10^6^) B cell precursors, and subsequently primed the recipients with the prior mRNA-LNP cocktail of N332-GT5, ApexGT5, and eOD-GT8 60mer, with the addition of mRNA-LNPs encoding a soluble 24mer called 10E8-GT12 (2 μg each). 10E8-GT12 has been further optimized from the 10E8-GT10 series for binding of both mature 10E8 and its precursors (28) and binds the HuGL18 H/L pair with 33 nM affinity (fig. S14A).

**Figure 8.**
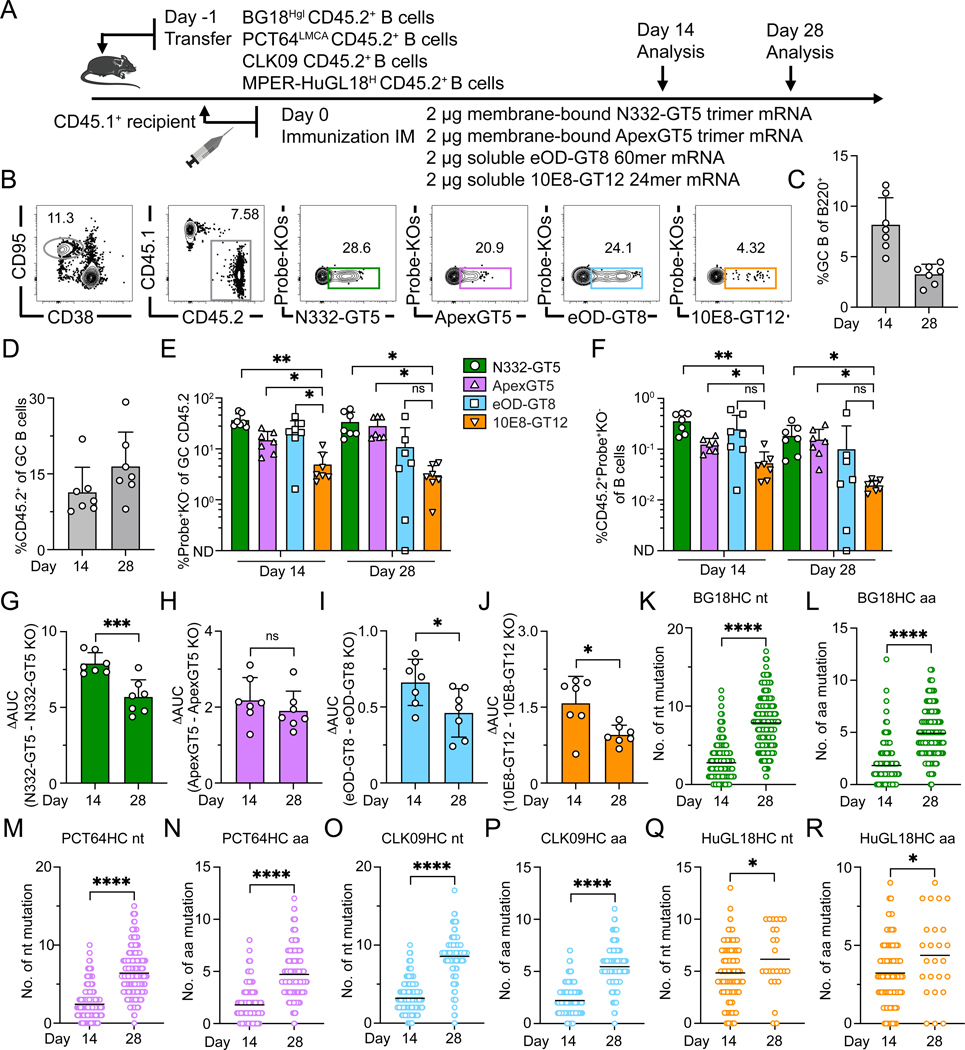
Simultaneous activation of four distinct classes of bnAb precursors. (**A**) Schematic showing naïve CD45.2 BG18^Hgl^ (post-transfer frequency = 7:10^6^), PCT64^LMCA^ (post-transfer frequency = 10:10^6^), CLK09 (post-transfer frequency = 20:10^6^), and HuGL18^H^ (post-transfer frequency = 100:10^6^) B cell adoptive transfer recipients immunized with quadruple mRNA-LNP immunogens (N332-GT5 trimer, ApexGT5 trimer, eOD-GT8 60mer, and 10E8-GT12 24mer) simultaneously via IM injection. Samples were collected at 14 and 28 dpi. Data was collected from a single run. (**B**) Gating strategy plots showing GC, CD45.2 cells in GC, N332-GT5, ApexGT5, eOD-GT8 and 10E8-GT12 epitope-specific binders among GC CD45.2 cells after immunization. (**C–F**) Frequencies of GC B cells (C), CD45.2^+^ B cells in GC (D), V3-glycan epitope specific (N332-GT5^+^KO^−^), Apex-specific (ApexGT5^+^KO^−^), CD4bs-specific (eOD-GT8^+^KO^−^) and MPER-specific (10E8-GT12^+^KO^−^) binders of GC CD45.2^+^ cells (E) gated as in (B), and CD45.2 epitope specific binders in total B cells (F) 14 and 28 dpi; n=7 in each condition. Statistics generated using Brown-Forsythe and Welch’s ANOVA with Dunnett’s T3 multiple comparison in (E & F). Not detected (ND). Bars indicate mean + SD. Not significant (ns); *P < 0.05; **P < 0.01. (**G–J**) ELISA quantification of N332-GT5-specific, ApexGT5-specific, eOD-GT8-specific and 10E8-GT12-specific IgG 14 and 28 dpi. ΔAUC comparison of titers for N332-GT5 and N332-GT5-KO (G); ApexGT5 and ApexGT5-KO (H) eOD-GT8 and eOD-GT8-KO (I); 10E8-GT12 and 10E8-GT12-KO (J). Each symbol represents a different mouse with 7 mice in each condition. Statistics generated using t test. Bars indicate mean + SD. Not significant (ns); *P < 0.05; ***P < 0.001. (**K–R**) Mutations across all sites of BCR heavy chains sequenced from CD45.2^+^Probe^+^KO^−^IgD^−^ B cells 14 and 28 dpi. gl BG18 HC nt (K); gl BG18 HC aa (L); LMCA PCT64 HC nt (M); LMCA PCT64 HC aa (N); CLK09 HC nt (O); CLK09 HC aa (P); HuGL18^H^ HC nt (Q); HuGL18^H^ HC aa (R). Statistics generated using Mann-Whitney test. Bars indicate mean. *P < 0.05; ****P < 0.0001.

Samples were assayed 14 and 28 dpi (Fig. 8A). Co-administration of the quadruple-GT mRNA-LNP cocktail induced strong GC reactions and a fraction of CD45.2^+^ cells participating in GCs comparable to prior mRNA-LNP combinations (Fig. 8, B to D). CD45.2 epitope-specific binders targeting all antigens were prevalent at both time points, although the fraction of GC CD45.2 binders or CD45.2 binders in total B cells specific for 10E8-GT12 was lower than for the other three epitopes (Fig. 8, E and F). Serum IgG against each antigen was detected (fig. S14, B to I), but the epitope-specific ΔAUC of N332-GT5 was, as in previous mixtures, highest (Fig. 8, G to J). Additionally, 10x BCR sequencing found that all lineages accumulated HC SHM over time (Fig. 8, K to R), and BG18, PCT64 and CLK09 precursors acquired similar hotspot mutation patterns as observed in single or combination activations described above (fig. S14, J to L). HuGL18 exhibited high mutation rates in HCDRs (fig. S14M), and all lineages underwent extensive diversification (fig. S14N). Generally, sequences recovered at day 28 remained distant from the mature bnAbs, though key or on-track mutations were observed (fig. S15). The addition of 10E8-class/MPER-directed bnAb precursors to the repertoire and a 10E8-GT immunogen to the cocktail did not inhibit the development of the other three lineages, suggesting that cocktails may be flexible as novel GT priming immunogens become available.

## Discussion

The enormous global diversity of HIV-1 is a major challenge for vaccine development (31). The outcomes of passive protection trials in NHPs (64, 65) and clinical trials (35) suggests that antibody-mediated protection may best be accomplished by engaging multiple targets on Env. The SARS-CoV2 pandemic brought mRNA-LNP vaccine technologies to the fore, and previous work in similar systems suggested that mRNA-LNP delivered membrane-anchored ApexGT5 could lower the activation threshold for PCT64^LMCA^ B cells (25). mRNA-LNP immunogens have also been developed to elicit BG18-class (21), VRC01-class (19, 20), and 10E8-class bnAb precursor responses (28), though not all perform a similar membrane display. Here, we found that a cocktail combining mRNA-LNP immunogens encoding the two membrane-anchored trimers ApexGT5 and N332-GT5, and the two soluble nanoparticles eOD-GT8 60mer and 10E8-GT12 24mer, could elicit precursors to PCT64-class/V2-apex, BG18-class/V3-glycan, VRC01-class/CD4bs, and 10E8-class/MPER bnAbs simultaneously, paving the way for the development of a multi-epitope HIV vaccine delivered in a logistically feasible number of immunizations.

A major challenge presented by combined protein immunization lies in formulation. Choice of adjuvant, for example, may substantially affect antigen presentation (66) and thus the efficacy of GT priming immunogens (29). The lack of a similar need for adjuvant selection and a comparatively simple immunization regimen may be major practical benefits of mRNA-LNP cocktails. Delivery by mRNA-LNP also allows for membrane-anchored presentations of antigen not feasible with protein. Immunization experiments with ApexGT6 soluble and membrane-bound mRNA-LNPs suggest that membrane-bound presentation may underpin improved PCT64^LMCA^ activation after the delivery of the triple mRNA cocktail versus the triple soluble protein cocktail. Notably, EMPEM also found off-target serum binding to the gp41 base after soluble protein immunization but not after the mRNA cocktail. While improved B cell receptor activation by membrane-anchored immunogens is supported by prior research on the immune synapse (67), this difference may potentially be driven by the specific binding modality of V2-apex bnAbs.

Immunogen concentration and availability over time modulates the GC reaction (68–70), and more is not always better, as recently demonstrated with ChAdOx1 nCov-19, where a lower dose prime was more efficient in clinic trials (71). By restricting antigen availability, a lower dose may select for fewer but higher affinity B cells, while a high dose may reduce selection stringency, resulting in diverse GC B cells of a broad range of affinities (72). The highest mRNA dose we used elicited larger GCs at day 14, but most cells found in those GCs at all doses were CD45.1, which are non-specific responses that were increasing alongside specific responses. The highest dose did, however, also have a larger proportion of CD45.2^+^ cells retained in the GC at day 28, indicating that a broader pool of bnAb precursor candidates may be available for later boost stages. To link our finding that bnAb precursors to all three epitopes could be efficiently stimulated and acquire similar SHM in the tested timeframe across a range of mRNA-LNP doses to antigen availability is, however, complicated by the complexity of connecting mRNA dose to the amount of antigen ultimately produced. Nevertheless, the ability to maintain efficacy at lowered combined mRNA doses may benefit clinical translation.

While increased initial PCT64^LMCA^ frequencies improved the response to the ApexGT5 protein trimer at day 14, this was no longer the case by day 28, and increased PCT64^LMCA^ precursor frequencies of the same magnitude did not affect GC expansion or SHM dynamics after mRNA-LNP co-immunization. BG18^Hgl^ was minimally affected by 10-fold frequency changes after either protein or mRNA-LNP co-immunization. In contrast, initial clonotype frequency has been previously observed as an important determinant of GC dynamics in VRC01-class precursor lines (50, 59, 73). Variable sensitivity to starting frequency may be the result of differential antibody feedback kinetics in the VRC01-class CLK lines as opposed to BG18^Hgl^ and PCT64^LMCA^: epitope-specific IgG antibody levels against the N332-GT5 and ApexGT5 trimers mounted rapidly relative to specific IgG levels against eOD-GT8 after mRNA-LNP immunization in our adoptive transfer models. Serum antibodies can affect naïve B cell GC entry and residency by forming immunocomplexes that lower the threshold for low affinity naïve B cell to enter GCs or, conversely, masking the epitopes to block naïve B cells (74); the latter effect has been observed with N332-GT immunogens (40). Therefore, epitope-specific high affinity antibodies in serum may block further naïve BG18^Hgl^ and PCT64^LMCA^ precursors from entering ongoing GCs via a stronger antibody feedback mechanism than occurs in CLK09. Part of the difference may lie in the use of trimers: ApexGT5 and N332-GT5 are relatively small and low avidity and thus may reach their maximum response over a narrower precursor frequency range than the 60mer nanoparticle. Alternatively, or additionally, we noted that during N332-GT5 and ApexGT5 mRNA co-immunization, a very high proportion of GCs were occupied by CD45.2 B cells: this may have saturated the antigen presentation capacity of the follicular dendritic cells or monopolized T cell help, rendering the system inflexible to increased precursor inputs at the frequencies tested.

In summary, we found that, in a multi-epitope activation scenario, membrane-bound mRNA-LNP immunogens could lower the activation threshold for bnAb precursors recalcitrant to protein immunization. Furthermore, we demonstrated that a cocktail of GT mRNA-LNP immunogens—some of which are now in clinical trials—drove a balanced precursor response. However, while protein trimer immunogens failed to efficiently elicit PCT64^LMCA^ in the dual immunization experiment conditions here, despite evidence of Sigma adjuvant being effective with ApexGT5 in the same knockin mouse model (25), that does not rule out the possibility that an alternative formulation may have rescued the response. Recent studies in NHPs found that the related protein trimer immunogen ApexGT6 efficiently activates PCT64-class bnAb precursors when delivered by mRNA-LNP or as bolus adjuvanted protein with Saponin/MPLA nanoparticles (SMNP) (75) and even when delivered as SMNP-adjuvanted protein alongside N332-GT5 trimer and a 10E8-GT12 24mer (76), although the escalating dose immunization used in in the Sutton et al. study may not be directly comparable to the bolus approach deployed here. A further limitation on the interpretative strength of the protein elicitation is that some of the protein priming experiments were only performed once, as were the quadruple mRNA cocktail prime and the direct comparison of soluble vs. membrane-anchored Apex-GT6 mRNA-LNP. Nonetheless, the efficacy of multi-priming in both NHPs (76) and knockin mouse models, and the activation in vivo here of BCRs with sequences found in the human repertoire, suggest that these findings can inform human regimens. The maintenance of existing responses when additional mRNA-LNPs were added also suggests that a priming cocktail may be readily adapted to the incorporation of new immunogens to additional classes as they are developed. Thus, GT priming immunogen cocktails could be developed to initiate development of multiple classes of bnAbs, allowing for a simplified immunization schedule for a multi-epitope HIV-1 vaccine using the single-epitope GT immunogens closest to the clinic.

## Materials and Methods

### Study Design

The primary objective of this study was to determine whether HIV bnAb precursors to distinct Env epitopes can be elicited simultaneously. We designed experiments using our previously described mouse models bearing human bnAb precursors and applied well-characterized GT protein and mRNA-LNP immunogens. After vaccination, we performed experiments using flow cytometry, cell sorting, single-cell 10X sequencing, ELISA, EMPEM and pseudo-virus neutralization assays to address this question. Mice were randomly allocated to experimental groups, but investigators were not blinded to the groups. All animals were included in all analysis except a single mouse with poor lymphocyte viability in fig. S4. The sample size and the number of experimental replicates are reported in the figure legends and selected on the basis of previous studies using these models and similar or identical immunogens. Reporting on animal experiments informed by ARRIVE guidelines.

Suppliers and catalogue numbers for all key resources found below are available in Table S1.

### Mouse models and subject details

Healthy adult male or female mice heterozygous for the BG18^Hgl^, PCT64^LMCA^, CLK09 and MPER-HuGL18^H^ knockins were generated as previously reported (25, 28, 29, 49, 50) and maintained in the C57BL/6J (CD45.2+/+) background. 8–12-week-old male B6.SJL-Ptprca Pepcb/BoyJ mice (CD45.1+/+) purchased from the Jackson laboratory were used in this study. Mice were housed at the animal facility of Mass General Hospital (MGH), with free access to food and water, controlled temperature and a 12:12 h light-dark cycle. The mouse maintenance and experiments were performed following the approved protocols by the Institutional Animal Care and Use Committee (IACUC) of MGH, an Association for Assessment and Accreditation of Laboratory Animal Care (AAALAC) International-accredited facility, under Animal Study Protocols 2016N000286 and 2016N000022.

### B cell adoptive transfer

Adoptive transfer performed as previously described in (21). Lymphocytes from male or female BG18^Hgl^, PCT64^LMCA^, CLK09 and HuGL18^H^ mice were isolated from spleens. After collection, spleens were crushed in FACS buffer (DPBS containing 2% FBS) and filtered with a 70 μm cell strainer, after which the cell suspension was subjected to pan B isolation kit (Miltenyi Biotec) according to the manufacturer’s protocol. Finally, the B cell pellets were suspended in DPBS. Cell viability and concentration were analyzed by the cell counter (LUNA-FL^™^), then naive B cells were transferred to male CD45.1^+^ recipient mice at the specified frequencies via intravenous (IV) injection. The BG18^Hgl^, PCT64^LMCA^, CLK09 and HuGL18^H^ cells were transferred at the default frequencies of 7:10^6^, 10:10^6^, 20:10^6^ and 100:10^6^, respectively, as previously reported (14, 20, 21, 25, 28, 29, 50), except for the precursor frequency assays in Figures 2 and 4, in which precursor frequencies of 70:10^6^ and 100:10^6^ were produced for BG18^High^ and PCT64^High^ groups.

### Immunization

Immunization performed as previously described in (21). Protein immunogens, described previously [N332-GT5 (21), ApexGT5 (25), eOD-GT8 60mer (19), 10E8-GT12 (28)], were diluted in PBS to reach a volume of 100 μl/per mouse and were mixed with Sigma adjuvant dissolved in PBS at the volume of 100 μl/per mouse. The immunogen/adjuvant complex was then shaken on a shaking table for 30–40 min before each mouse was given 200 μl immunogen/adjuvant complex via intraperitoneal (IP) injection from both sides of the abdomen. mRNA lipid nanoparticle (LNP) immunogens (19, 21, 25, 27, 28) were diluted in PBS to reach a volume of 100 μl/per mouse. Each mouse was given 100 μl mRNA-containing mixture via intramuscular (IM) injection in the thigh muscles of the two side hind limbs. The immunogen dosages used are noted in the schematic graphs in each figure.

### Protein production for ELISA and sorting probes

His-tagged and His-Avi-tagged trimeric (N332-GT5 (21), ApexGT5 (25)) and monomeric eOD-(GT8 (50), and 10E8-GT12 (28)) antigens, were produced by transient transfection of HEK-293F cells (Thermo Fisher) and purified by affinity chromatography (IMAC) using HisTrap excel columns (Cytiva) followed be size-exclusion chromatography (SEC) using either Superdex 75 10/300 GL (eOD-GT8 and 10E8-GT12) or Superdex 200 Increase 10/300 GL (N332-GT5 and ApexGT5) columns (Cytiva). The molecular weight and the homogeneity of the antigens were confirmed by size-exclusion chromatography-multi-angle light scattering (SEC-MALS) in phosphate-buffered saline (PBS) using Superdex 75 10/300 GL or Superdex 200 Increase 10/300 GL columns (Cytiva) columns operating with an isocratic flow of 0.5 mL/minute followed by DAWN HELEOS II and Optilab T-rEX detectors (Wyatt Technology). His-Avi-tagged antigens were biotinylated using BirA (Avidity) and re-purified to remove excess biotin using either Superdex 75 10/300 GL or Superdex 200 Increase 10/300 GL columns (Cytiva) (19). Biotinylated monomeric probes were pre-reacted with fluorescently labeled streptavidin (SA-488, SA-647, SA-594, SA-BV421, SA-BV605 or SA-BUV395) in a 4:1 molar ratio in independent tubes for at least 30 min and then combined with fluorescently labeled antibodies for flow cytometry and cell sorting staining.

### Flow cytometry

Flow cytometry performed as previously described in (21). Single-cell suspensions were generated by mechanical dissociation of spleen (after protein immunization) or inguinal, popliteal and iliac lymph nodes (after mRNA-LNP immunization). ACK buffer (Lonza Bioscience) was used to remove red blood cells and then cell pellets were suspended in fluorescence-activated cell sorting (FACS) buffer (DPBS containing 2% FBS). Cell viability was stained with Live/Dead Blue (Invitrogen) in PBS (100 μl, 1:1000 dilution) at 4°C for 10 min. Samples were centrifuged at 400 × g for 3 min at 4°C before supernatant was discarded, and then cells were Fc blocked (clone 2.4G2, BD Biosciences) in FACS buffer (100 μl, 1:100 dilution) at 4°C for another 10 min. After centrifugation and removal of the supernatant, cells were preincubated with freshly fluorescently labeled protein probes (100 μl, 40 μM) for 20 min before the cocktail of antibodies (100 μl, 1:100 dilution) against surface markers merged and stained for another 20 min at 4°C. The antibody panel included Alexa Fluor 700-CD3, Alexa Fluor 700-F4/80, Alexa Fluor 700-Gr1, BV786-B220, BV510-CD38, PE-Cy7-CD95, PerCP-Cy5.5-CD45.1, PE-CD45.2, PE-Dazzle594-CD138, APC-Cy7-IgM and BV650-IgD. Afterward, samples were washed once, suspended in FACS buffer and filtered with 70 μm cell strainer before flow cytometry recording. Samples were acquired and events were recorded on a BD LSR Fortessa for flow cytometry analysis. Data was analyzed by FlowJo software (Tree Star, Inc.). B cells were gated as single cell/live dead Blue^−^/B220^+^Dump^−^ (CD3, F4/80 and Gr1). The strategy for gating B cell subsets was shown in the representative FACS plots of each figure. To quantify the percentage of CD45.2 binders in total B cells, numbers from relevant gates (CD45.2^+^CD45.1^−^/probe^+^probe-KO^−^) under B cells were multiplied out.

### Cell sorting

Cell sorting performed as previously described in (21). Spleens after protein immunization, or inguinal, popliteal, and iliac lymph nodes after mRNA-LNP immunization were collected from CD45.1^+^ recipient mice and crushed and filtered, then cell pellets were suspended in FACS buffer. Cells were Fc blocked (clone 2.4G2, BD Biosciences, 1:100 dilution) in FACS buffer for 10 min at 4°C. Cells were centrifuged at 400 × g for 3 min at 4°C before supernatant was discarded, then stained with fluorescently labeled probes (100 μl, 40 μM), in which streptavidin-Alexa fluor-488, streptavidin-Alexa fluor-647 were conjugated with all the WT probes and streptavidin-Alexa fluor-594 with all the KO probes for 20 min, and then incubated with a cocktail of antibodies against surface markers (100 μl, 1:100 dilution), and anti-mouse CD45 hashtag oligos (2 μl each) for 30 min at 4°C. Afterwards, cells were thoroughly washed and suspended in FACS buffer with DAPI (1:1000 dilution). Cell sorting was done on a BD FACS Aria Fusion or Aria II at a flow rate of <4000 events/second for tube sorting using a 70 μm nozzle. Sorting strategy was shown in Supplementary figure 2. Recorded events were analyzed by FlowJo software and sorted samples were used for the 10x Genomics single-cell experiments below.

### 10x Genomics single-cell BCR sequencing and analysis

BCR sequencing performed as previously described in (21, 29, 77). Briefly, less than 1500 targeted live cells were sorted from each sample and no more than 12,000 in total per reaction were encapsulated and converted into several DNA libraries following the 10x Next GEM Single cell 5’ protocol (10x Genomics). Single cells were isolated with Gel Bead-In-EMulsions (GEMs) using the Chromium controller provided by 10x Genomics, resulting in uniquely barcoded transcriptome for each individual cell. After initial cDNA amplification and conversion to dsDNA, individual sequencing libraries were generated for gene expression, V(D)J repertoires and hashtag oligos. Library quality was analyzed using a TapeStation 4200 (Agilent). Libraries were pooled at a ratio based on depth requirements established by 10x genomics and subsequently sequenced using a NextSeq 2000 sequencer (Illumina).

Raw base call files generated by sequencing were demultiplexed, aligned and aggregated using the pipeline offered as part of Cell Ranger v8 (10x Genomics). For V(D)J repertoire analysis, immunoglobulin V genes were determined by Cell Ranger followed by a customized analysis pipeline that includes the BG18 iGL heavy chain sequence, PCT64 LMCA heavy and light chain sequences, CLK09 germline heavy and light chain sequences and HuGL18 germline heavy chain sequence.

### ELISA

ELISA performed as previously described in (21). Serum antigen specific antibody titers were detected by ELISA, using anti-His Ab (1 μg/ml, 50 μl/well) overnight coating at 4 °C to capture His-tagged N332-GT5 (1 μg/ml, 50 μl/well) and ApexGT5 (2 μg/ml, 50 μl/well) WT or respective KO trimer antigen on the 96-well plate (Corning, REF 3690). N332-GT5 antigens were coated at 4°C overnight, while ApexGT5 antigens were coated at 4°C for 2h. His-tagged eOD-GT8 and 10E8-GT12 monomer WT or KO protein (1 μg/ml, 50 μl/well) was coated at 4°C overnight on the 96-well plate directly without anti-His Ab pre-coating. Antigen coated plates were blocked with 3% BSA (dissolved in 0.1% tween plus PBS) for 1h at room temperature (RT), then serially diluted mouse sera were incubated for two hours, and alkaline phosphatase conjugated anti-mouse IgG (Jackson ImmunoResearch, #115–055-071) at a 1:1000 dilution was incubated for another hour. p-Nitrophenyl phosphate (Sigma, # N2770) dissolved in ddH_2_O (50 μl/well, RT, 30 min) was used for coloration. Coloration was stopped with 2 mol/L NaOH (50 μl/well). Absorbance at 405 nm was determined with a plate reader (BioTek). Titers were determined from the dilution curve in the linear range of absorbance. ELISA curves were calculated and analyzed using GraphPad Prism (GraphPad).

### Electron microscopy polyclonal epitope mapping (EMPEM)

Serum was pooled from mice that were primed with triple GT mRNA-LNP immunogens (high dose group: 10 μg each) at day 28, or with triple protein immunogens (5 μg N332-GT5, 5 μg ApexGT5, and 10 μg eOD-GT8 60mer) at day 28. Serum processing and sample preparation to obtain polyclonal fabs for electron microscopy were previously described in (78). Briefly, IgG was isolated using Protein G (Cytiva). Papain (Sigma Aldrich) was used to digest IgG to fabs. A cocktail consisting of 5 μg each of eOD-GT8, N332-GT5 and ApexGT5 was mixed with 1 mg of Fab mixture (containing Fc and residual papain) for an overnight incubation and the complex was then purified using a Superdex 200 Increase 10/300 GL gel filtration column (Cytiva).

Purified complexes were concentrated and diluted to a final concentration of 0.02 mg/mL. The diluted samples were deposited on glow-discharged carbon-coated copper mesh grids, followed by staining with 2% (w/v) uranyl formate. Electron microscopy images were collected on an FEI Tecnai Spirit T12 equipped with an FEI Eagle 4k x 4k CCD camera (120 keV, 2.06 Å/pixel) using the Leginon suite (79) and processed using Relion 3.1 (80) following standard 2D and 3D classification procedures. The best 3D class from each dataset was refined. UCSF ChimeraX (81) was used to generate the composite maps, and the refined 3D maps have been deposited to the Electron Microscopy Data Bank.

### TZM-bl pseudovirus neutralization assay

Pseudoviruses (PSV)were produced in HEK293T cells (RRID:CVCL_0063, ATCC Cat# CRL 3216) co-transfected using FuGENE 6 (Promega, cat# E2691) or PEI Max (Polisciences, cat #24765–1) with pseudovirus Env-expressing plasmid and Env-deficient backbone plasmid (PSG3DEnv). Pseudoviruses were harvested 72 hours post-transfection, sterile filtered (0.45 μm), and concentrated (EMD Millipore, Cat# UFC905024). Mouse serum IgGs were purified according to manufacturer’s protocol (Thermo Scientific, Cat# 45204). Equal volumes (15 μl) of serially diluted purified IgG at a starting concentration of 50 μg/ml were incubated with 15 μl HIV pseudovirus in round bottom 96-well plate (Costar, Cat# 3788) at 37°C for 1 hour. TZM-bl cells (RRID:CVCL_B478, NIH AIDS Cat # 8129) were seeded 24 hours prior at 100,000 cells/ml in 50 μl/well of half-area 96-well plates (Costar, Cat # 3688). Plates were removed from the 37°C incubator, culture media aspirated and 25 μl of mix of mAb +PSV (with or without DEAE-Dextran(Spectrum Chemical, Cat# DE-132–25GM) (5 μg/ml final concentration) were added to each well and incubated at 37°C for 24 hours in a humidified atmosphere of 5% CO_2_. After 24 hours incubation, 75 μl of culture media was added. Plates were incubated in same conditions for additional 48 hours. After 48 hours (total 72 hours), culture media was removed, and cells were lysed with 45 μl/well 1x Luciferase Culture Lysis buffer (Promega Cat# E1531) for 20 min at RT. Neutralization was measured by adding 30 μl luciferase reagent/well (Promega, Cat #E1501) and measuring luminescence. IC_50_ was calculated using a nonlinear regression curve fit, sigmoidal, 4PL equation constrained from 0–100% in GraphPad Prism 10.4.2.

### Phylogenetic analysis

BG18^Hgl^, PCT64^LMCA^, CLK09 and HuGL18 heavy chain nucleotide sequences from 10x single-cell BCR sequencing were aligned using MUSCLE. The genetic distance was modeling by Jukes-Cantor and tree was built by Neighbor-joining assay. The original gl BG18 (55), LMCA PCT64 (56), CLK09 (62) and HuGL18 (29) reference sequences were used as the starting nodes for tree plots. The clonal phylogeny tree plots were generated using Geneious Prime (Biomatters Ltd, New Zealand).

### Statistics

Where necessary for clarity, graphs were plotted on a log scale. The statistical analysis of data following a non-normal distribution was performed with non-parametric test, in which comparisons between two groups were made by Mann-Whitney and comparisons among more than two groups by Kruskal-Wallis. For data following a normal distribution, comparisons between two groups were assayed with Student’s t- test (equal variances) or Welch’s t-test (unequal variances); comparisons among more than two groups were assayed with One-Way ANOVA using Tukey’s multiple comparison (equal variances) or Brown-Forsythe and Welch ANOVA with Dunnett’s T3 multiple comparison (unequal variances). Statistical tests are described in their respective figure legends. P values were calculated using GraphPad Prism version 10.4.1. *P < 0.05; **P < 0.01; ***P < 0.001; ****P < 0.0001.

## Supplementary Material

data file S1

Supplemental Materials; Figure S1-S15 and Table S1 **Figure S1.** Quality control profile of ApexGT5 protein, related to Figure 1.
**Figure S2.** Cell sorting strategy for 10x, related to Figure 1.
**Figure S3.** BG18-class/V3-glycan and PCT64-class/V2-apex bnAb precursors can be simultaneously activated by mRNA-LNPs encoding two BG505 SOSIP MD39-derived trimers, related to Figure 3.
**Figure S4.** mRNA-LNP encoding membrane-bound ApexGT6 triggers stronger PCT64^LMCA^ activation than soluble formulation, related to Figure 3.
**Figure S5.** BG18-class/V3-glycan and PCT64-class/V2-apex bnAb precursor heavy chains simultaneously undergo SHM irrespective of initial precursor frequency, related to Figure 4.
**Figure S6.** Simultaneous activation of PCT64-class/V2-apex and VRC01-class/CD4bs bnAb precursors induces serum antibody response and precursor SHM, related to Figure 5.
**Figure S7.** BG18-class/V3-glycan bnAb precursors can also be activated in tandem with VRC01-class/CD4bs bnAb precursors, related to Figure 5.
**Figure S8.** BG18-class/V3-glycan and VRC01-class/CD4bs bnAb precursors undergo SHM simultaneously, related to Figure 5.
**Figure S9.** bnAb germline precursors to all three epitopes can be primed with variable efficacy by protein immunogens, related to Figure 6 and 7.
**Figure S10.** Endogenous CD45.1 activation after triple GT mRNA-LNP co-immunization, related to Figure 6.
**Figure S11.** Serum antibody response after triple mRNA-LNP co-administration, related to Figure 7.
**Figure S12.** BG18-class/V3-glycan, PCT64-class/V2-apex and VRC01-class/CD4bs bnAb precursors acquire SHM by a range of mRNA-LNP doses, related to Figure 7.
**Figure S13.** Neutralization potency of murine serum, related to Figure 7.
**Figure S14.** Simultaneous activation of four bNAb precursors elicited serum antibody responses and drove precursor SHM efficiently, related to Figure 8.
**Figure S15.** Amino acid sequence alignment of germline, mature and primed heavy chains, related to Figure 8.
**Table S1.** Key resources.

MDAR Reproducibility Checklist

## Data Availability

BG18^Hgl^, PCT64^LMCA^, CLK09 and HuGL18^H^ model animals are available from corresponding author F.D.B. (fbatista1@mgh.harvard.edu) on request, under a standard material transfer agreement with Massachusetts General Hospital. BCR sequences are affiliated with the BioProject PRJNA1284875 which has Targeted Locus Study project deposited at DDBJ/EMBL/GenBank under the accession KJBA00000000 and KJBB00000000. The version described in this paper is the first version, KJBA01000000 and KJBB00000000. Electron microscopy maps have been deposited to the Electron Microscopy Data Bank (EMDB) under accession codes EMD-70190 and EMD-70192. Plasmids or proteins related to the immunogens, sort reagents, or antibodies employed in this study are available from W.R.S. (schief@scripps.edu) under a material transfer agreement with The Scripps Research Institute. mRNA vaccine constructs may be made available from S.H. (Sunny.himansu@modernatx.com) if the recipient and Moderna are able to agree upon the terms of a material transfer agreement. Tabulated data underlying Figs. 1 to 8 and figs. S1, and S3 to S14 are provided in data file S1. All data are available in the main text or the supplementary materials.
