# Supplementary material for: Simultaneous priming of HIV broadly neutralizing antibody precursors to multiple epitopes by germline-targeting mRNA-LNP immunogens in mouse models
Source: Sci Immunol. Author manuscript; Available in PMC 2026 Jan 9. (PMC12784461; doi:10.1126/sciimmunol.adu7961)
Supplement: Supplemental Materials; Figure S1-S15 and Table S1 — Figure S1. Quality control profile of ApexGT5 protein, related to Figure 1. Figure S2. Cell sorting strategy for 10x, related to Figure 1. Figure S3. BG18-class/V3-glycan and PCT64-class/V2-apex bnAb precursors can be simultaneously activated by mRNA-LNPs encoding two BG505 SOSIP MD39-derived trimers, related to Figure 3. Figure S4. mRNA-LNP encoding membrane-bound ApexGT6 triggers stronger PCT64LMCA activation than soluble formulation, related to Figure 3. Figure S5. BG18-class/V3-glycan and PCT64-class/V2-apex bnAb precursor heavy chains simultaneously undergo SHM irrespective of initial precursor frequency, related to Figure 4. Figure S6. Simultaneous activation of PCT64-class/V2-apex and VRC01-class/CD4bs bnAb precursors induces serum antibody response and precursor SHM, related to Figure 5. Figure S7. BG18-class/V3-glycan bnAb precursors can also be activated in tandem with VRC01-class/CD4bs bnAb precursors, related to Figure 5. Figure S8. BG18-class/V3-glycan and VRC01-class/CD4bs bnAb precursors undergo SHM simultaneously, related to Figure 5. Figure S9. bnAb germline precursors to all three epitopes can be primed with variable efficacy by protein immunogens, related to Figure 6 and 7. Figure S10. Endogenous CD45.1 activation after triple GT mRNA-LNP co-immunization, related to Figure 6. Figure S11. Serum antibody response after triple mRNA-LNP co-administration, related to Figure 7. Figure S12. BG18-class/V3-glycan, PCT64-class/V2-apex and VRC01-class/CD4bs bnAb precursors acquire SHM by a range of mRNA-LNP doses, related to Figure 7. Figure S13. Neutralization potency of murine serum, related to Figure 7. Figure S14. Simultaneous activation of four bNAb precursors elicited serum antibody responses and drove precursor SHM efficiently, related to Figure 8. Figure S15. Amino acid sequence alignment of germline, mature and primed heavy chains, related to Figure 8. Table S1. Key resources. [file NIHMS2119667-supplement-Supplemental_Materials__Figure_S1-S15_and_Table_S1.pdf]

Science
AAAS

Supplementary Materials for

Simultaneous priming of HIV broadly neutralizing antibody precursors to multiple epitopes by germline-targeting mRNA-LNP immunogens in mouse models

Zhenfei Xie, Xuesong Wang, Yu Yan, Jon M. Steichen, Krystal M. Ma, Christopher A. Cottrell, Eleonora Melzi, Maria Bottermann, Paula Maldonado Villavicencio, Kimmo Rantalainen, Torben Schiffner, John E. Warner, Stephanie R. Weldon, Thavaleak Prum, Jordan R. Ellis-Pugh, Jonathan L. Torres, Abigail M. Jackson, Claudia T. Flynn, Gabriel Ozorowski, Sunny Himansu, Andrea Carfi, Andrew B. Ward, Usha Nair, William R. Schief, Facundo D. Batista

Corresponding authors: schief@scripps.edu (W.R.S.); fbatista1@mgh.harvard.edu (F.D.B.)

The PDF file includes:

Figs. S1 to S15
Table S1

Other Supplementary Material for this manuscript includes the following:

Data file S1
MDAR Reproducibility Checklist

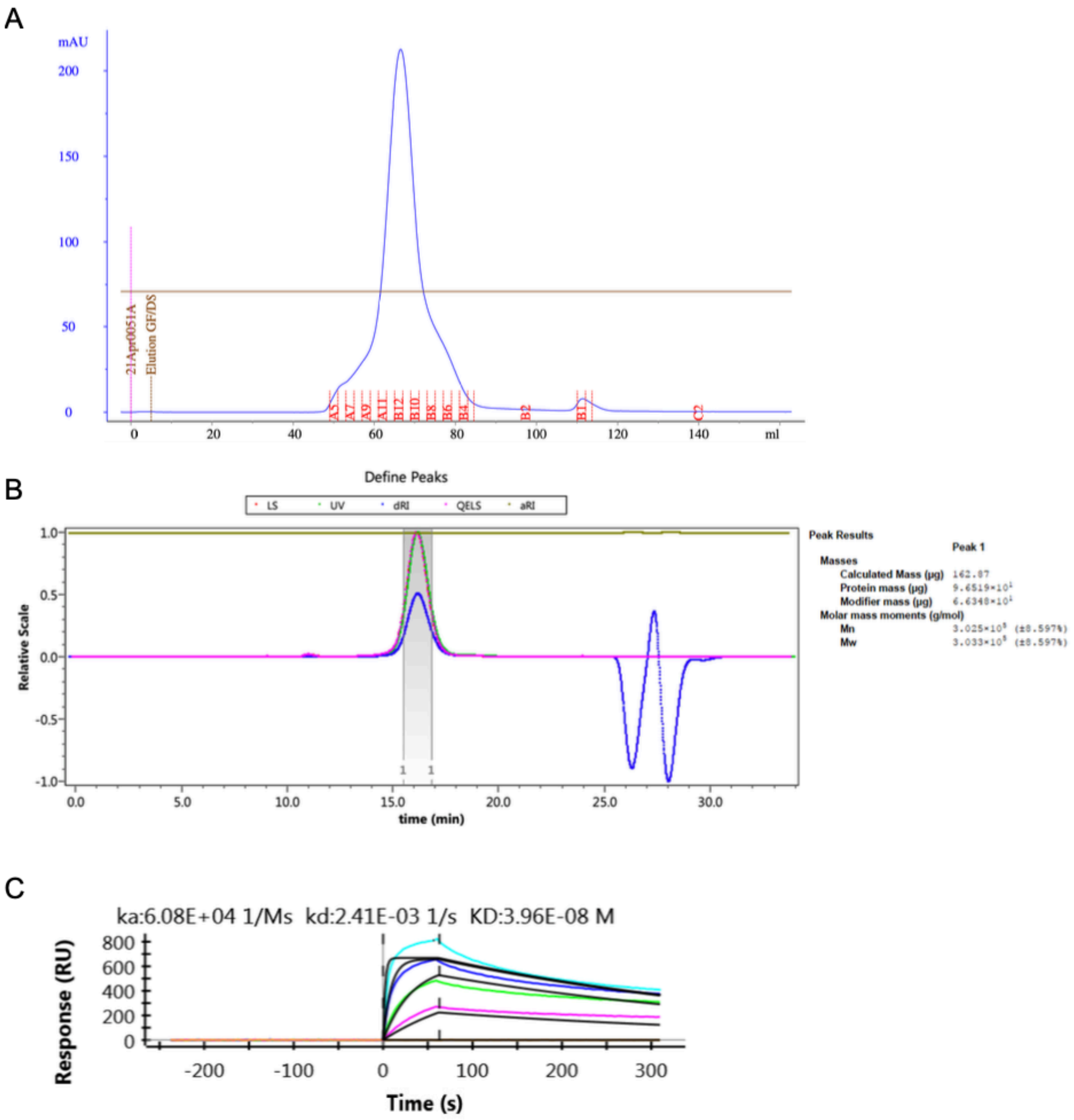

Figure S1. Quality control profile of ApexGT5 trimer protein, related to Figure 1.

(**A**) Size-exclusion traces of ApexGT5 immunogen after lectin purification show the construct elutes primarily as a single peak at B12. DNA was transfected with a plasmid into FreeStyle 293F cells and proteins were expressed at 37°C for six days. ApexGT5 protein trimers were purified

through lectin affinity and followed by size-exclusion chromatography with Superdex 200 increase column.

(**B**) SECMALS analysis of B12 in Fig. S1A confirms it is a trimer, with an observed molecular weight of 3.033×10^5 g/mol, which agrees with the expected trimeric molecular weight of 2.11×10^5 g/mol plus additional mass from native glycans.

(**C**) SPR sensorgram evaluating the binding of ApexGT5 to PCT64 LMCA: PCT64 ^{LMCA} IgG was captured as the ligand, with ApexGT5 trimer serving as the analyte. Analyte concentrations tested were: 7.5, 1.875, 0.4688, and 0.1172 μM. The data were fitted using a 1:1 binding model, where colored curves represent experimental data and the black curves show the fitted lines.

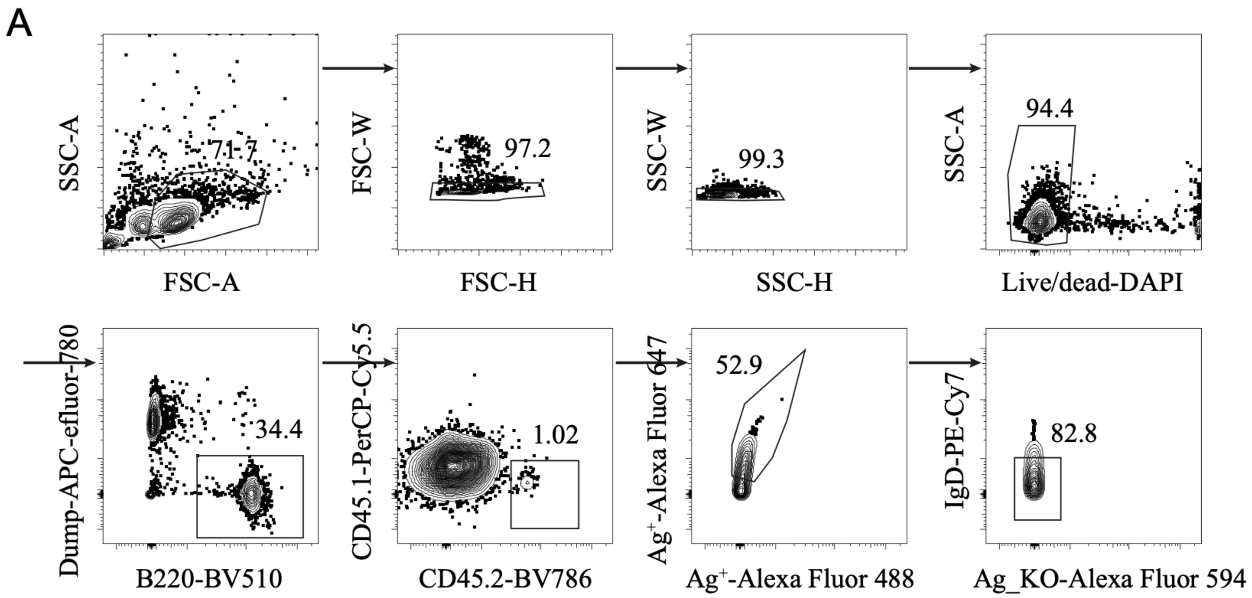

Figure S2. Cell sorting strategy for 10x, related to Figure 1.

(**A**) Gating strategy of cell sorting to isolate CD45.2$^+$ epitope-specific B cell antigen (Ag) binders (Single cell/Live/B220$^+$Dump (CD3, F4/80, Gr1)$^-$/CD45.2$^+$CD45.1$^-$/Ag$^+$/Ag_KO$^-$IgD$^-$) after immunization for 10x BCR sequencing.

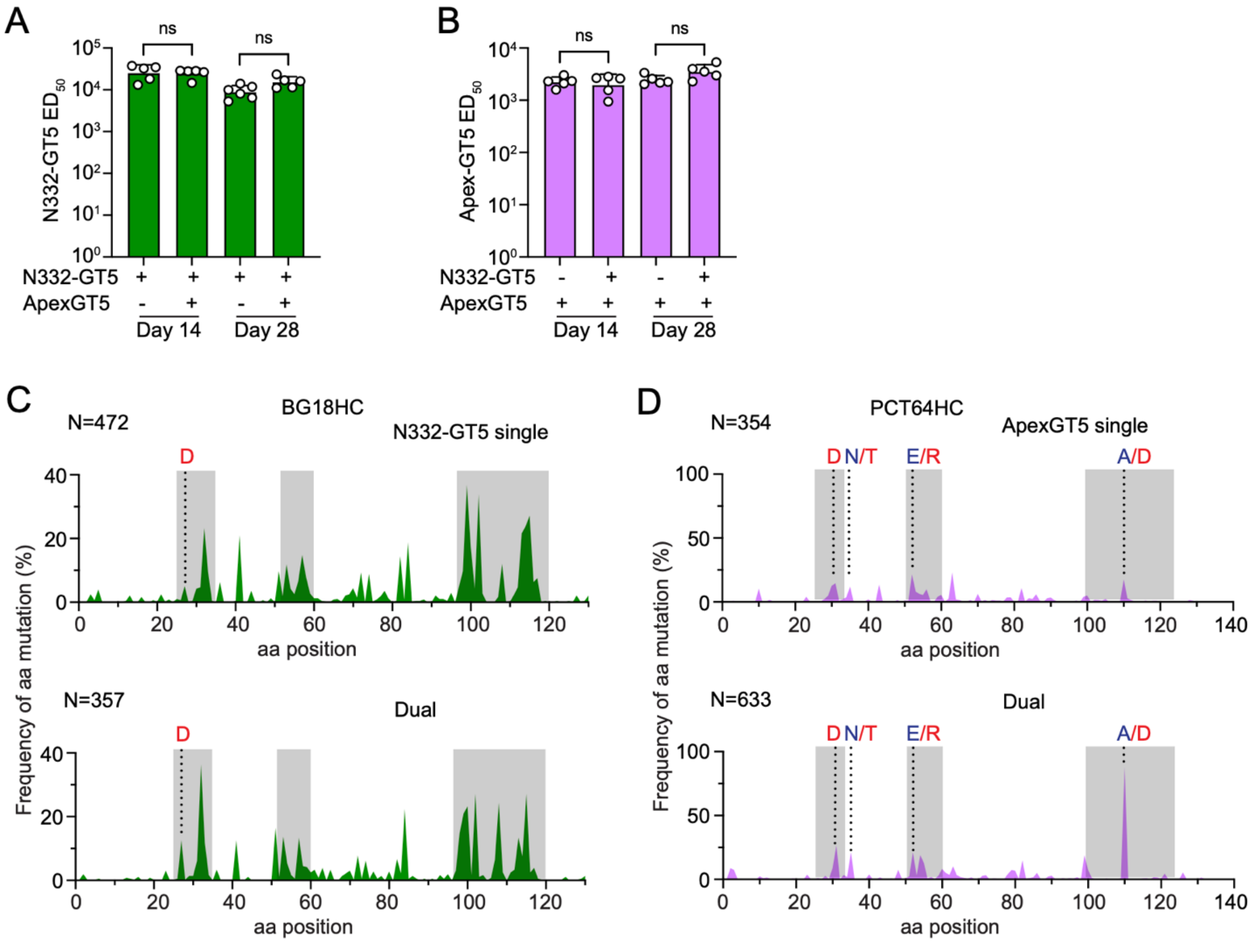

Figure S3. BG18-class/V3-glycan and PCT64-class/V2-apex bnAb precursors can be simultaneously activated by mRNA-LNPs encoding two BG505 SOSIP MD39-derived trimers, related to Figure 3.

(**A and B**) Serum IgG titers. Serum IgG ELISA 50% equilibrium dilution (ED_{50}) values for N332-GT5 (A) and ApexGT5 (B) 14 and 28 dpi. Each symbol represents a different mouse. n=5–6 in each condition. Statistics generated using one-way ANOVA with Tukey's multiple comparison test. Bars indicate geometric mean + geometric SD. Not significant (ns).

(**C and D**) Per site heavy chain aa mutation frequency 28 dpi. CDRs boxed in grey; key mature (red) and on-track (dark blue) mutations marked with letters. N at top left indicates sequences analyzed.

gl BG18 HC: N332-GT5 single prime (top); N332-GT5 + ApexGT5 dual prime (bottom) (C). LMCA PCT64 HC: ApexGT5 single prime (top); N332-GT5 + ApexGT5 dual prime (bottom) (D).

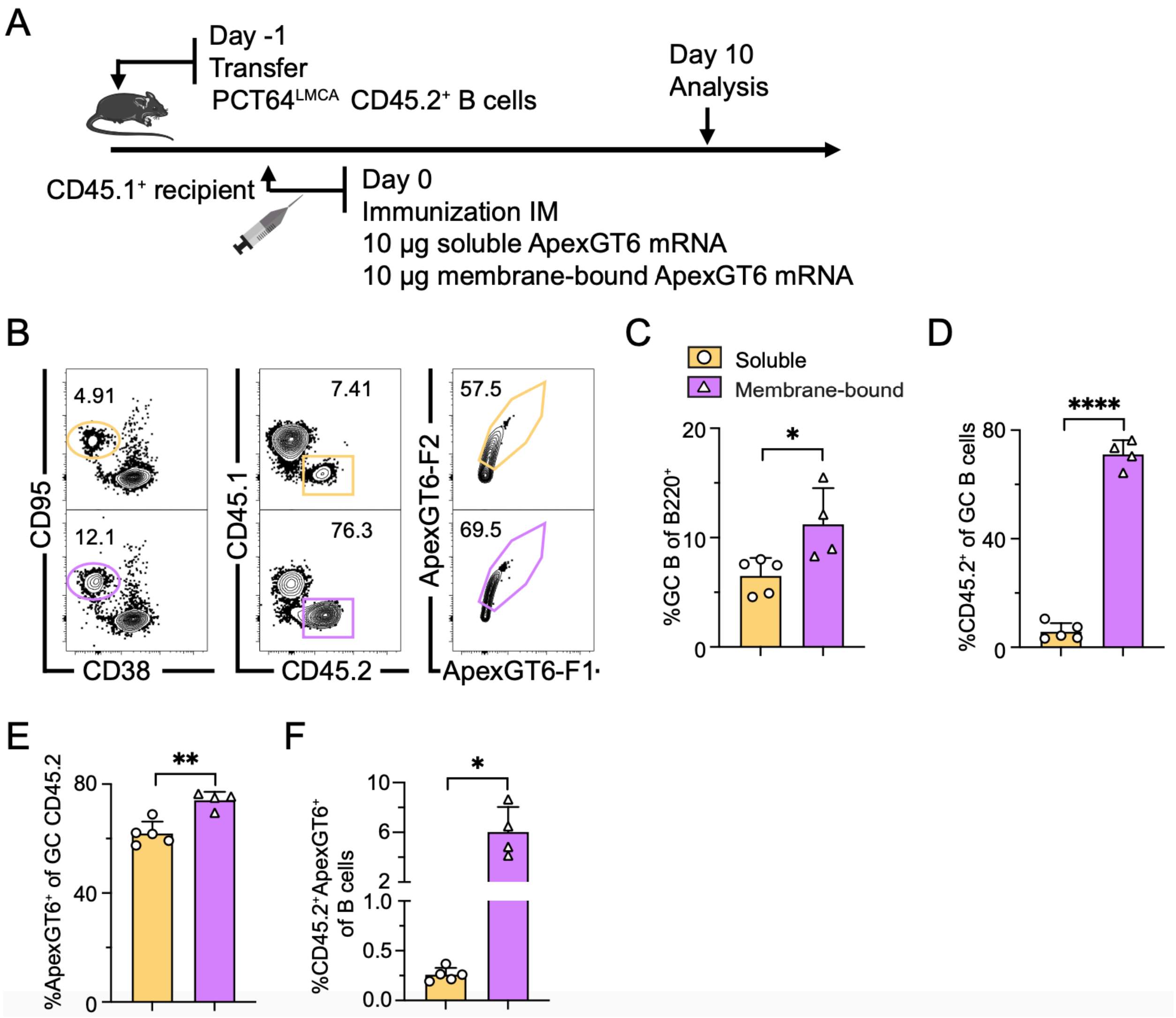

Fig S4. mRNA-LNP encoding membrane-bound ApexGT6 triggers stronger PCT64^{LMCA} activation than soluble formulation, related to Figure 3.

(**A**) Schematic showing naïve CD45.2^{+} PCT64^{LMCA} B cells (post-transfer frequency = 20:10^{6}) adoptively transferred into CD45.1 WT recipient (day -1), which were then primed with either soluble or membrane-bound ApexGT6 mRNA-LNP. Samples were collected 10 dpi. A sample with poor viability was excluded from the analysis. Data from a single run shown.

(**B**) Gating strategy showing GC, CD45.2 cells in GC, ApexGT6 antigen binders among GC CD45.2 cells post immunization.

(**C to F**) Frequencies of GC B cells (C), $CD45.2^+$ cells in GC (D), ApexGT6 binders among GC $CD45.2^+$ cells (E) gated as in (B), and $CD45.2^+$ ApexGT6 binders in total B cells (F) in each condition. Each symbol represents a different mouse; n=5 (Soluble); n=4 (Membrane-bound). Statistical analysis was performed using t test. Bars indicate mean + SD. *$P < 0.05$, **$P < 0.01$, ****$P < 0.0001$.

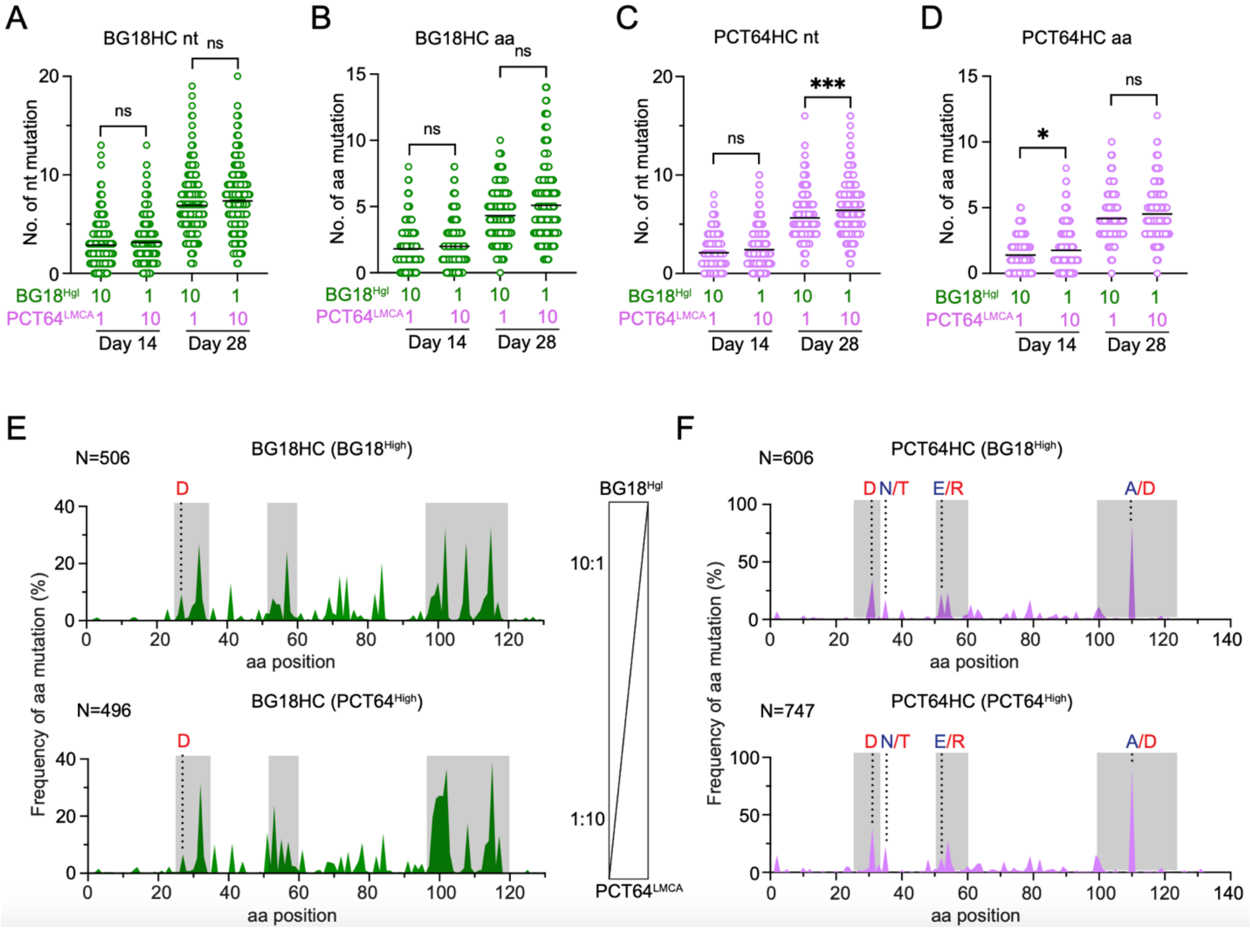

Figure S5. BG18-class/V3-glycan and PCT64-class/V2-apex bnAb precursor heavy chains simultaneously undergo SHM irrespective of initial precursor frequency, related to Figure 4.

(**A to D**) Heavy chain nt and aa mutation number across all sites of BCRs isolated from CD45.2^{+} Probe^{+}KO^{-}IgD^{-} B cells 14 and 28 days after immunization as in Fig. 4A. BG18 HC nt (A); BG18 HC aa (B); PCT64 HC nt (C); PCT64 HC aa (D). Statistical analysis was performed using Kruskal-Wallis test. Bars indicate mean. Not significant (ns); **P* < 0.05, ****P* < 0.001.

(**E and F**) Per site heavy chain aa mutation frequency 28 dpi. CDRs boxed in grey; key mature (red) and on-track (dark blue) mutations marked with letters. N at top left indicates number of sequences in analysis. gl BG18 HC: BG18^{High} (top); PCT64^{High} (bottom) (E). LMCA PCT64 HC: BG18^{High} (top); PCT64^{High} (bottom) (F).

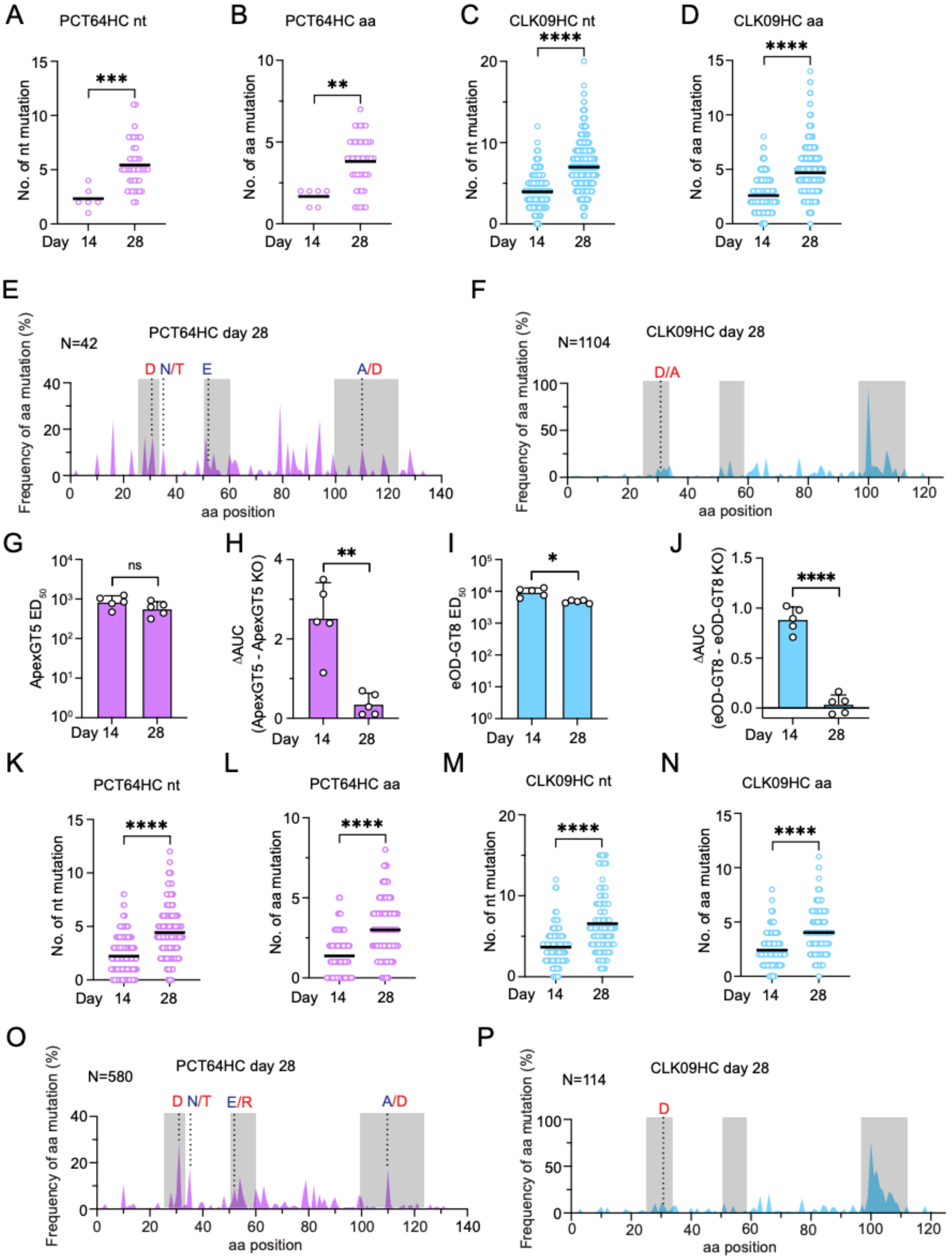

Figure S6. Simultaneous activation of PCT64-class/V2-apex and VRC01-class/CD4bs bnAb

precursors induces serum antibody response and precursor SHM, related to Figure 5.

(**A to D**) Number of nt and aa mutations across all heavy chain sites after protein immunization. Sequences isolated from CD45.2$^+$Probe$^+$KO$^-$IgD$^-$ B cells 14 and 28 dpi as in Fig. 5A. LMCA PCT64 HC nt (A); LMCA PCT64 HC aa (B); CLK09 HC nt (C); CLK09 HC aa (D). Statistics generated using Mann-Whitney test. Bars indicate mean. $**P < 0.01$; $***P < 0.001$; $****P < 0.0001$.

(**E and F**) Per site heavy chain aa mutation frequency 28 dpi by protein immunogens. CDRs boxed in grey; key mature (red) and on-track (dark blue) mutations marked with letters. N at top left indicates sequences in analysis. LMCA PCT64 HC (E); CLK09 HC (F).

(**G to J**) Serum IgG antibody titer measurement. Serum IgG ELISA ED_{50} values for ApexGT5 (G) or eOD-GT8 (I) 14 and 28 dpi of mRNA-LNPs. ΔAUC comparison of ApexGT5 and ApexGT5 KO (H) eOD-GT8 and eOD-GT8 KO (J). Each symbol represents a different mouse with five mice at both time points. Statistics generated using Student's t test (G and J) or Welch's t test (H and I). Bars represent geometric mean + geometric SD (G and I) or indicate mean + SD (H and J). Not significant (ns); $*P < 0.05$; $**P < 0.01$; $****P < 0.0001$.

(**K to N**) Number of nt and aa mutations across all heavy chain sites after mRNA-LNP immunization. Sequences isolated from CD45.2$^+$Probe$^+$KO$^-$IgD$^-$ B cells 14 and 28 dpi as in Fig. 5G. LMCA PCT64 HC nt (K); LMCA PCT64 HC aa (L); CLK09 HC nt (M); CLK09 HC aa (N). Statistics generated using Mann-Whitney test. Bars indicate mean. $****P < 0.0001$.

(**O and P**) Per site heavy chain aa mutation frequency 28 dpi of mRNA-LNPs. CDRs boxed in grey; key mature (red) and on-track (dark blue) mutations marked with letters. N at top left indicates sequences in analysis. LMCA PCT64 HC (O); CLK09 HC (P).

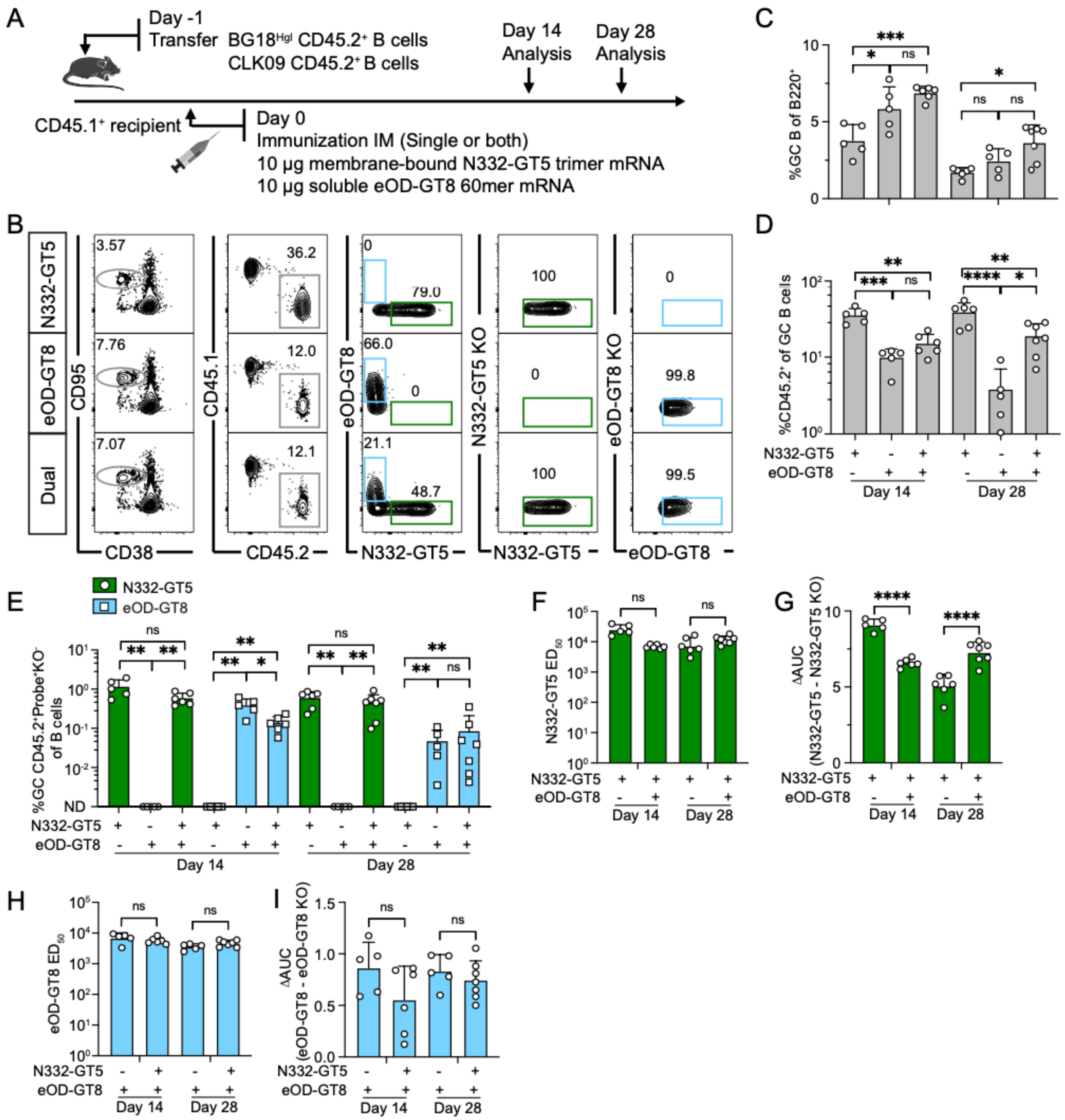

Figure S7. BG18-class/V3-glycan bnAb precursors can also be activated in tandem with VRC01-class/CD4bs bnAb precursors, related to Figure 5.

(**A**) Schematic of experimental approach: adoptive transfer of naïve CD45.2 BG18^{Hgl} (post-transfer frequency = 7:10^6) and CLK09 (post-transfer frequency = 20:10^6) B cells into CD45.1 WT mice (day -1) followed by immunization of recipients with either N332-GT5 trimer mRNA-LNP, eOD-GT8

60mer mRNA-LNP, or both via IM injection (day 0). Samples were assayed 14 and 28 dpi. Data collected from a single run.

(**B**) Gating strategy plots showing GC, CD45.2 cells in GC, N332-GT5- and eOD-GT8-binders within the GC CD45.2 fraction and binding specificity after either single or dual mRNA-LNP immunization.

(**C to E**) Frequencies of GC B cells, $CD45.2^+$ B cell in GC, V3-glycan epitope specific ($N332\text{-}GT5^+KO^-$) and CD4bs epitope-specific ($eOD\text{-}GT8^+KO^-$) GC $CD45.2^+$ cells in total B cells in each condition 14 and 28 dpi; n=5–7 in each group. Statistical analysis was performed using One-Way ANOVA with Tukey's multiple comparison test in (C and D); Mann-Whitney or t test in (E). Not detected (ND). Bars indicate mean + SD. Not significant (ns); *$P < 0.05$; **$P < 0.01$; ***$P < 0.001$; ****$P < 0.0001$.

(**F to I**) Serum IgG antibody titer measurement. Serum IgG ELISA ED_{50} values for N332-GT5 (F) or eOD-GT8 (H) 14 and 28 dpi. ΔAUC comparison of N332-GT5 and N332-GT5 KO (G) eOD-GT8 and eOD-GT8 KO (I). Each symbol represents a different mouse; 5–7 mice per group. Statistical analysis was performed using one-way ANOVA with Tukey's multiple comparison test. Bars indicate geometric mean + geometric SD (F and H) or mean + SD (G and I). Not significant (ns); ****$P < 0.0001$.

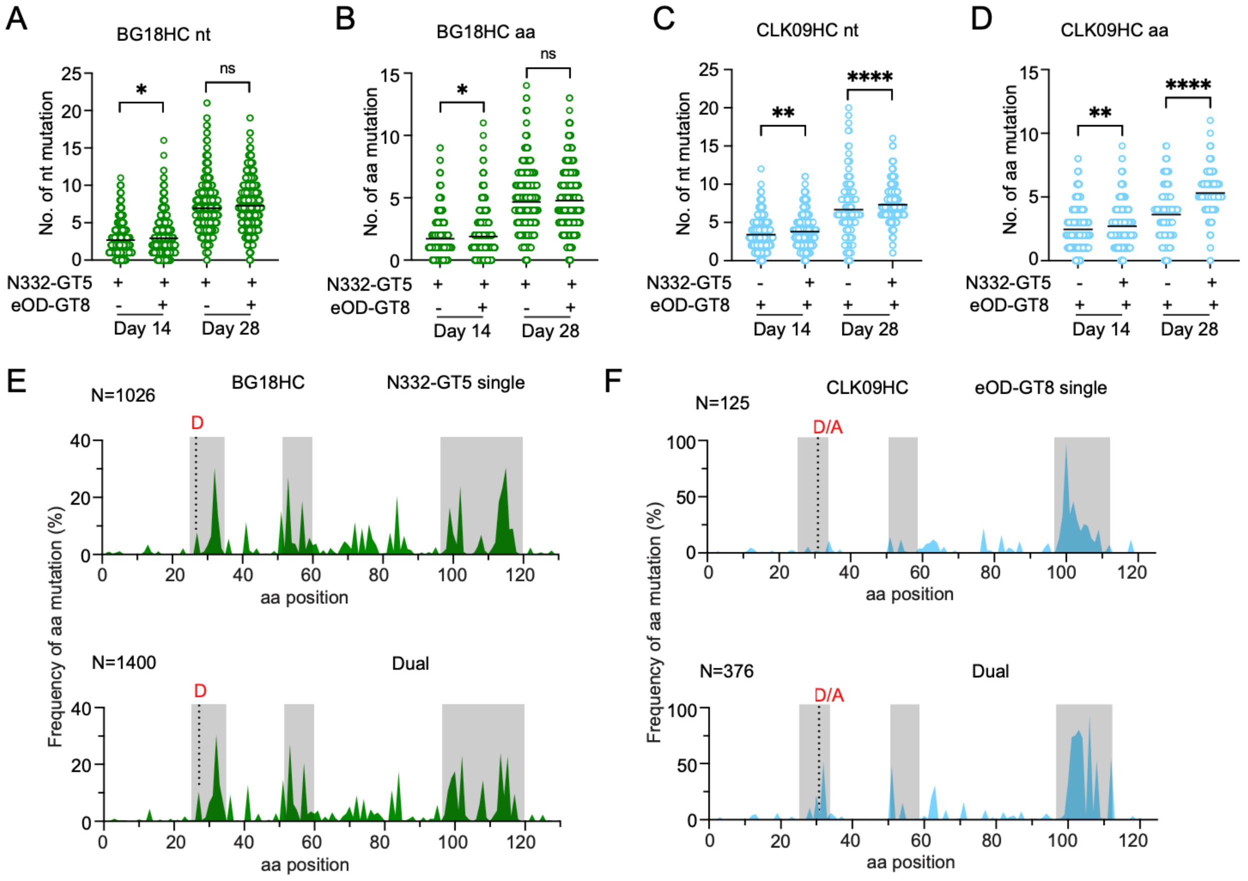

Figure S8. BG18-class/V3-glycan and VRC01-class/CD4bs bnAb precursors undergo SHM simultaneously, related to Figure 5.

(**A to D**) Number of heavy chain nt and aa mutations across all sites of BCRs isolated from CD45.2^{+}Probe^{+}KO^{-}IgD^{-} B cells 14 and 28 dpi. gl BG18 HC nt (A); gl BG18 HC aa (B); CLK09 HC nt (C); CLK09 HC aa (D). Statistical analysis was made using Kruskal-Wallis test. Bars indicate mean. Not significant (ns); *P < 0.05; **P < 0.01; ****P < 0.0001.

(**E and F**) Per site heavy chain aa mutation frequency 28 dpi. CDRs boxed in grey; key mature mutations marked by red letters. N at top left indicates sequences included in the analysis. gl BG18 HC: N332-GT5 single prime (top); N332-GT5 + eOD-GT8 dual prime (bottom) (E). CLK09 HC:

eOD-GT8 single prime (top); N332-GT5 + eOD-GT8 dual prime (bottom) (F).

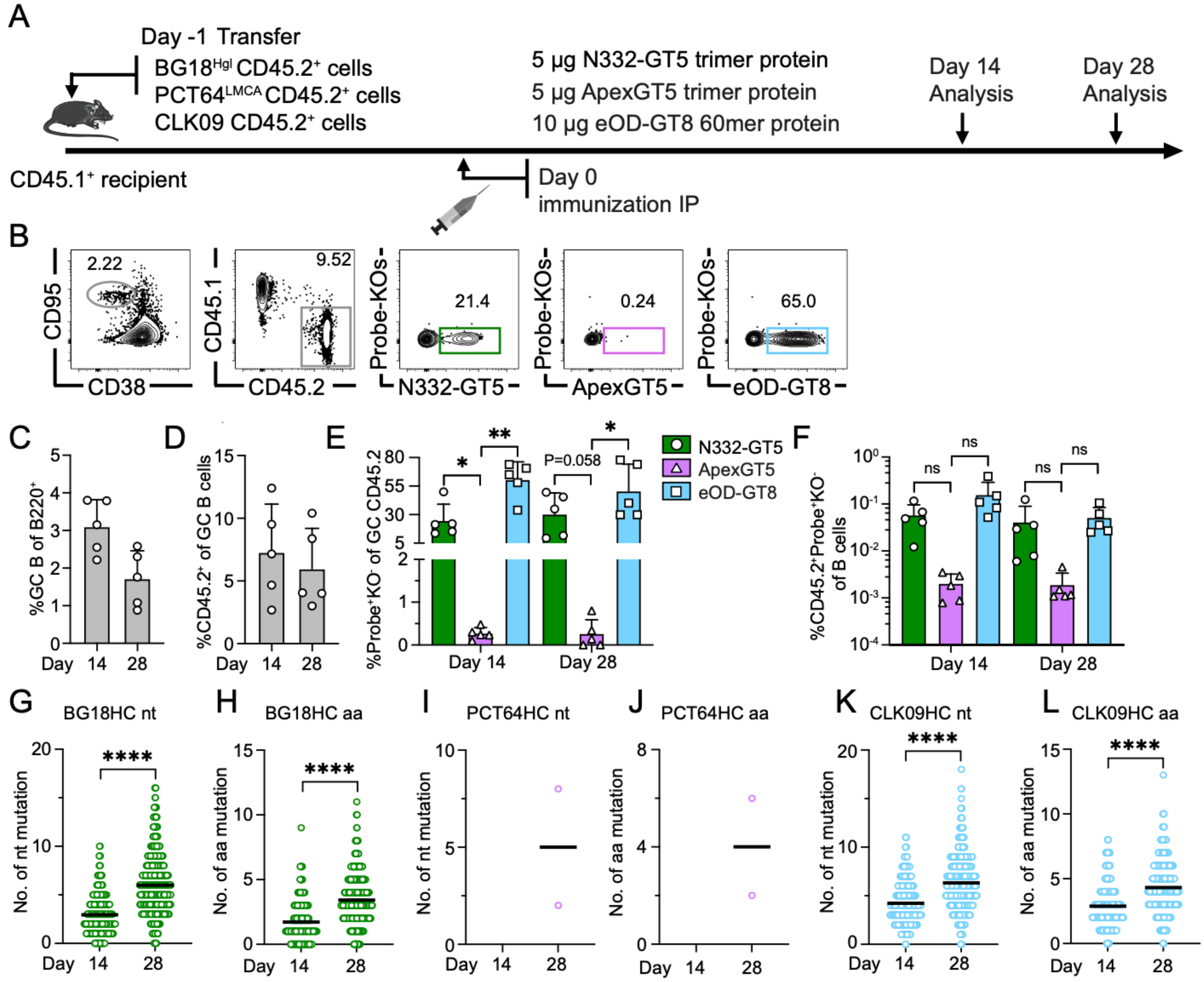

Figure S9. bnAb germline precursors to all three epitopes can be primed with variable efficacy by protein immunogens, related to Figure 6 and 7.

(**A**) Schematic showing naive CD45.2 BG18^{Hgl} (post-transfer frequency = 7:10^{6}), PCT64^{LMCA} (post-transfer frequency = 10:10^{6}), and CLK09 (post-transfer frequency = 20:10^{6}) B cells adoptively transferred into shared CD45.1 WT hosts (day -1); recipients were immunized with N332-GT5 trimer, ApexGT5 trimer and eOD-GT8 60mer triple protein via IP injection (day 0). Samples were collected 14 and 28 dpi for analysis. Experiments were performed twice, and representative data from one are shown.

(**B**) Gating strategy plots showing GC, CD45.2 cells in GC, N332-GT5, ApexGT5 and eOD-GT8 epitope specific binders of CD45.2 cells in GC after immunization.

(**C to F**) Frequencies of GC B cells (C), CD45.2$^+$ B cell in GC (D), V3-glycan–epitope-specific (N332-GT5$^+$KO$^-$), apex-epitope-specific (ApexGT5$^+$KO$^-$) and CD4bs-specific (eOD-GT8$^+$KO$^-$) binders of GC CD45.2$^+$ cells (E) gated as in (B) and CD45.2 epitope specific binders in total B cells (F) 14 and 28 dpi; n=5 in each condition. Statistics generated using Brown-Forsythe and Welch's ANOVA with Dunnett's T3 multiple comparison in (E and F). Bars indicate mean + SD. Not significant (ns); **P* < 0.05; ***P* < 0.01.

(**G to L**) Heavy chain nt and aa mutation number across all sites of BCRs isolated from CD45.2$^+$Probe$^+$KO$^-$IgD$^-$ B cells 14 and 28 dpi. gl BG18 HC nt (G); gl BG18 HC aa (H); LMCA PCT64 HC nt (I); LMCA PCT64 HC aa (J); CLK09 HC nt (K); CLK09 HC aa (L). Note: LMCA PCT64 sequences were not recovered on day 14, and only two sequences were recovered on day 28. Statistics generated using Mann-Whitney test (G and H, K and L). Bars indicate mean. *****P* < 0.0001.

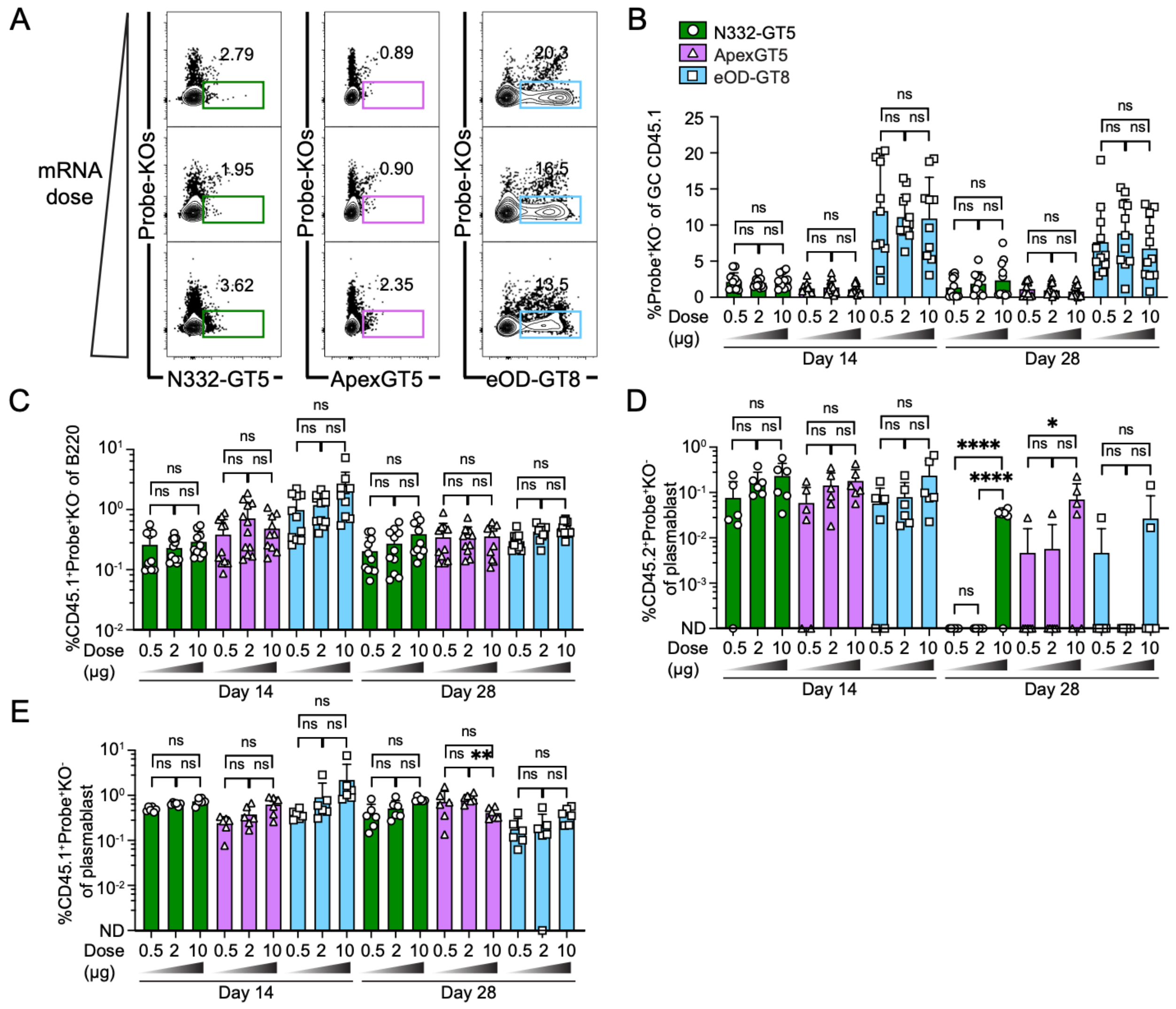

Figure S10. Endogenous CD45.1 activation after triple GT mRNA-LNP co-immunization, related to Figure 6.

(**A to E**) Analysis of additional cell populations from the immunization experiments presented in Figure 6.

(A) Representative FACS plots showing N332-GT5, ApexGT5 and eOD-GT8 epitope-specific binders among GC CD45.1 cells after immunization.

(B and C) Frequencies of V3-glycan epitope specific (N332-GT5$^+$KO$^-$), apex-specific (ApexGT5$^+$KO$^-$) and CD4bs-specific (eOD-GT8$^+$KO$^-$) binders of GC CD45.1 cells (B) gated as in (A), and CD45.1

epitope specific binders in total B cells (C) 14 and 28 dpi; n=10–12 in each condition as main Figure 6. Statistical analysis was performed using Brown-Forsythe and Welch ANOVA with Dunnett's T3 multiple comparison. Not detected (ND); Bars indicate mean + SD. Not significant (ns).

(**D and E**) Frequencies of V3-glycan epitope specific (N332-GT5^{+}KO^{-}), apex-specific (ApexGT5^{+}KO^{-}) and CD4bs-specific (eOD-GT8^{+}KO^{-}) binders from CD45.2^{+} (D) or CD45.1^{+} cells (E) in total plasmablast (CD138^{+}Dump (Gr1, CD3, F4/80)$^{-}$) 14 and 28 dpi. Data was shown from a single run. n=6 in each condition. Statistical analysis was performed using Brown-Forsythe and Welch ANOVA with Dunnett's T3 multiple comparison. Not detected (ND). Bars indicate mean + SD. Not significant (ns), **P* < 0.05; ***P* < 0.01; *****P* < 0.0001.

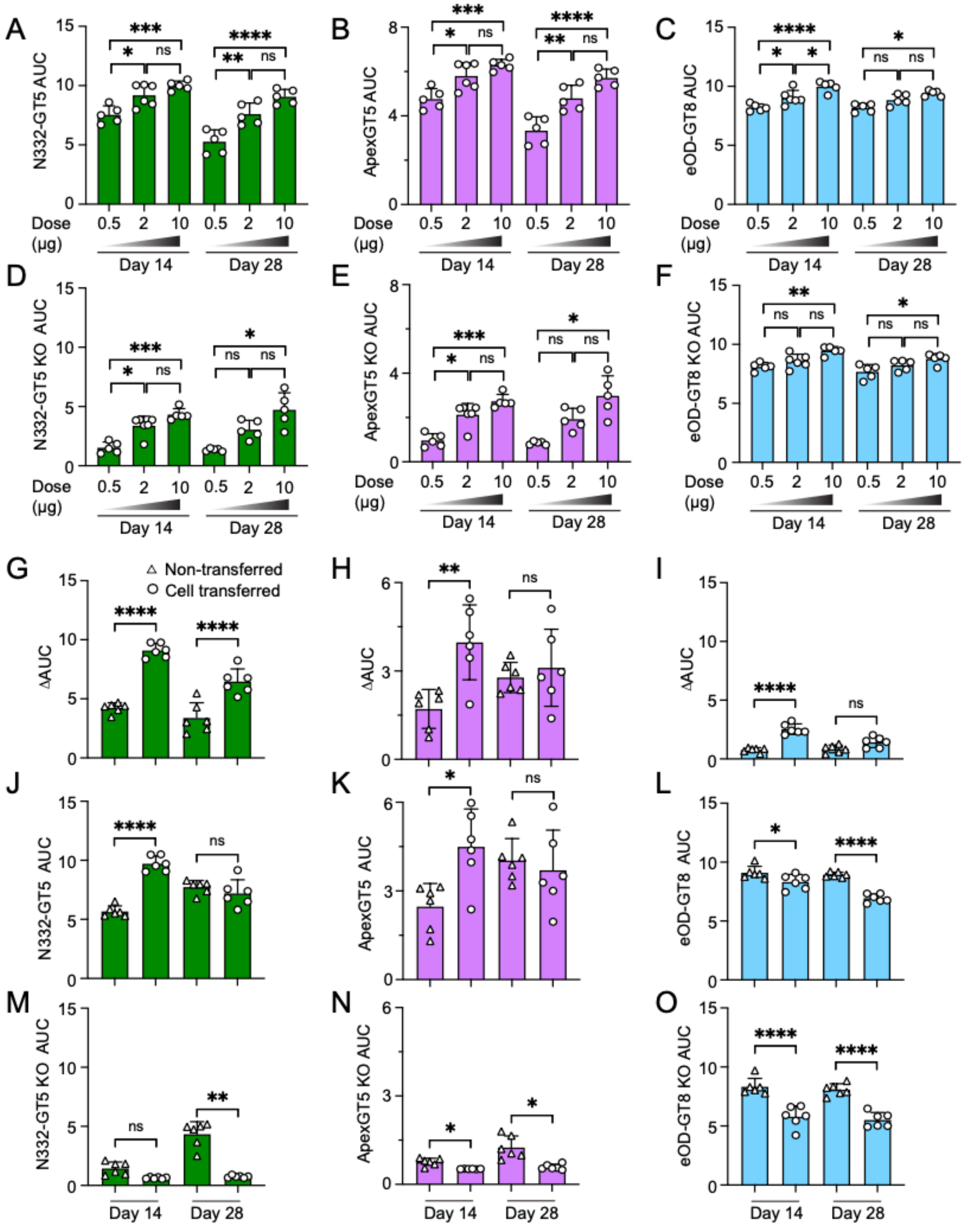

Figure S11. Serum antibody response after triple mRNA-LNP co-administration, related to Figure 7.

(**A to F**) ELISA quantification of serum IgG AUC values for WT proteins of N332-GT5 (A), ApexGT5

(B), eOD-GT8 (C) and epitope KO proteins of N332-GT5 (D), ApexGT5 (E) and eOD-GT8 (F) 14 and 28 dpi. Each symbol represents a different mouse. n=5–6 in each condition as in Fig. 7 (A to C). Statistics were generated using One-Way ANOVA with Tukey's multiple comparison test (A to C and F) and Brown-Forsythe and Welch's ANOVA with Dunnett's T3 multiple comparison (D and E). Bars indicate mean + SD. Not significant (ns); **P* < 0.05; ***P* < 0.01; ****P* < 0.001; *****P* < 0.0001. (**G to O**) ELISA quantification of serum IgG for each antigen protein in $CD45.2^+$ cells ($7:10^6$ for $BG18^{Hgl}$, $10:10^6$ for $PCT64^{LMCA}$ and $20:10^6$ for CLK09) adoptively transferred mice or *non-transferred* WT mice 14 and 28 dpi of intermediate dose of triple mRNA-LNPs (2 µg each). ΔAUC comparison of titers for N332-GT5 and N332-GT5-KO (G); ApexGT5 and ApexGT5 KO (H); eOD-GT8 and eOD-GT8 KO (I). AUC values for WT proteins of N332-GT5 (J); ApexGT5 (K); eOD-GT8 (L). AUC values for epitope KO proteins of N332-GT5 (M), ApexGT5 (N) and eOD-GT8 (O). n=6 in each condition. Statistics were generated using One-Way ANOVA with Tukey's multiple comparison test (G to L and O) and Brown-Forsythe and Welch's ANOVA with Dunnett's T3 multiple comparison (M–N). Bars indicate mean + SD. **P* < 0.05; ***P* < 0.01; *****P* < 0.0001.

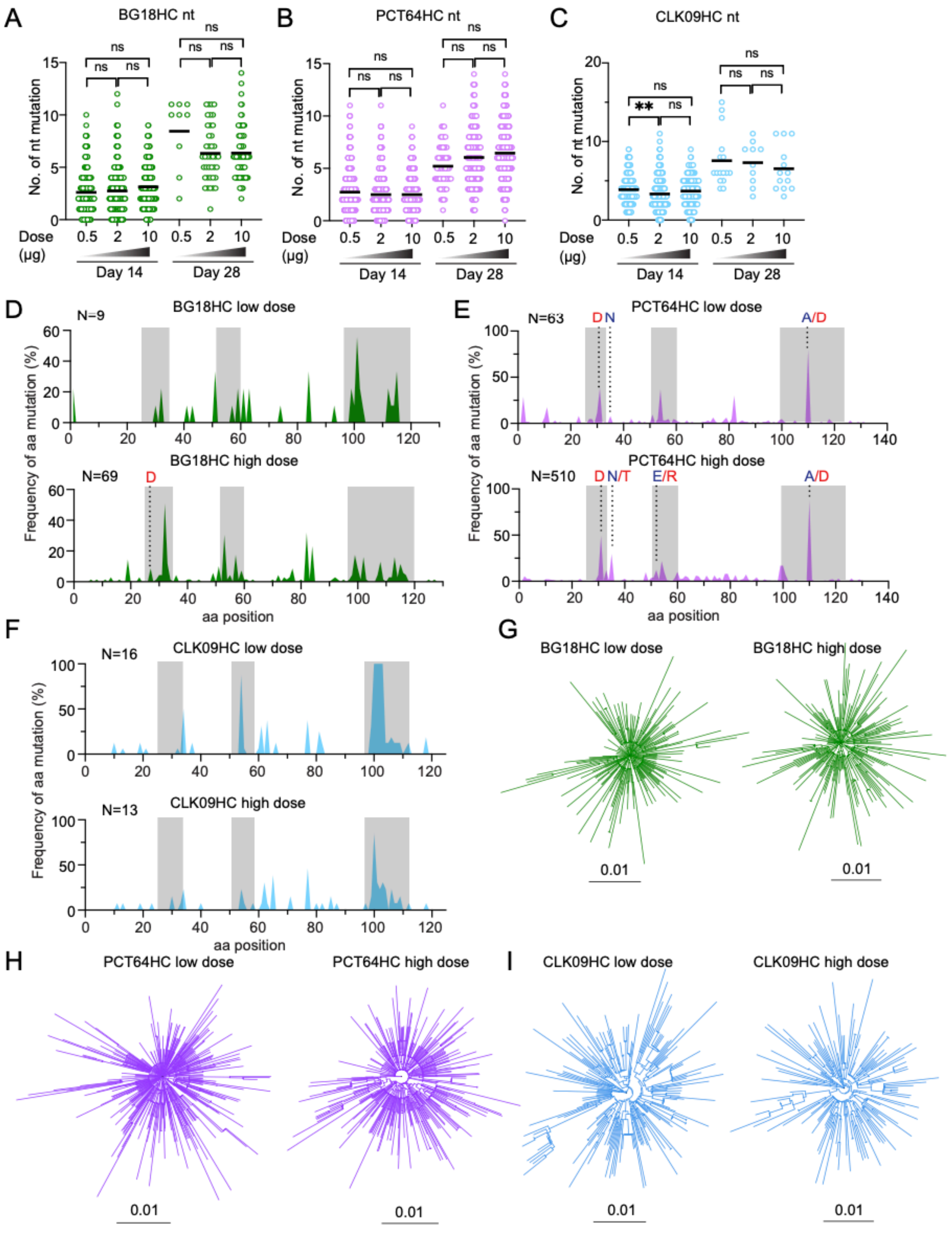
A
BG18HC nt
No. of nt mutation
Dose (µg)
0.5 2 10 0.5 2 10
Day 14
Day 28
ns
B
PCT64HC nt
C
CLK09HC nt
**
D
BG18HC low dose
N=9
Frequency of aa mutation (%)
BG18HC high dose
N=69
aa position
E
PCT64HC low dose
N=63
PCT64HC high dose
N=510
F
CLK09HC low dose
N=16
CLK09HC high dose
N=13
G
BG18HC low dose
BG18HC high dose
0.01
H
PCT64HC low dose
PCT64HC high dose
I
CLK09HC low dose
CLK09HC high dose

Figure S12. BG18-class/V3-glycan, PCT64-class/V2-apex and VRC01-class/CD4bs bnAb precursors acquire SHM by a range of mRNA-LNP doses, related to Figure 7.

(**A to C**) Mutations across all sites of BCR heavy chains sequenced from $CD45.2^{+}Probe^{+}KO^{-}IgD^{-}$ B cells 14 and 28 dpi. BG18 HC nt (A); LMCA PCT64 HC nt (B); CLK09 HC nt (C). Statistical analysis performed using Kruskal-Wallis test. Bars indicate mean. Not significant (ns); $^{**}P < 0.01$.

(**D to F**) Per site heavy chain aa mutation frequency 28 dpi. CDRs boxed in grey; key mature (red)—or for PCT64 only, on-track (dark blue) (*56*)—mutations marked with letters. N at top left indicates number of sequences included in analysis. gl BG18 HC (D); LMCA PCT64 HC (E); CLK09 HC (F). Low dose (top); high dose (bottom).

(**G to I**) Phylogenetic tree plots of each lineage after triple GT mRNA-LNP 14 dpi. Each tree denotes a clonal lineage. Branch lengths are scaled by estimated heavy chain nt mutations. gl BG18 HC (G); LMCA PCT64 HC (H); CLK09 HC (I). Low dose (left); high dose (right). Tree Scale (0.01) indicates the number of substitutions per site.

A

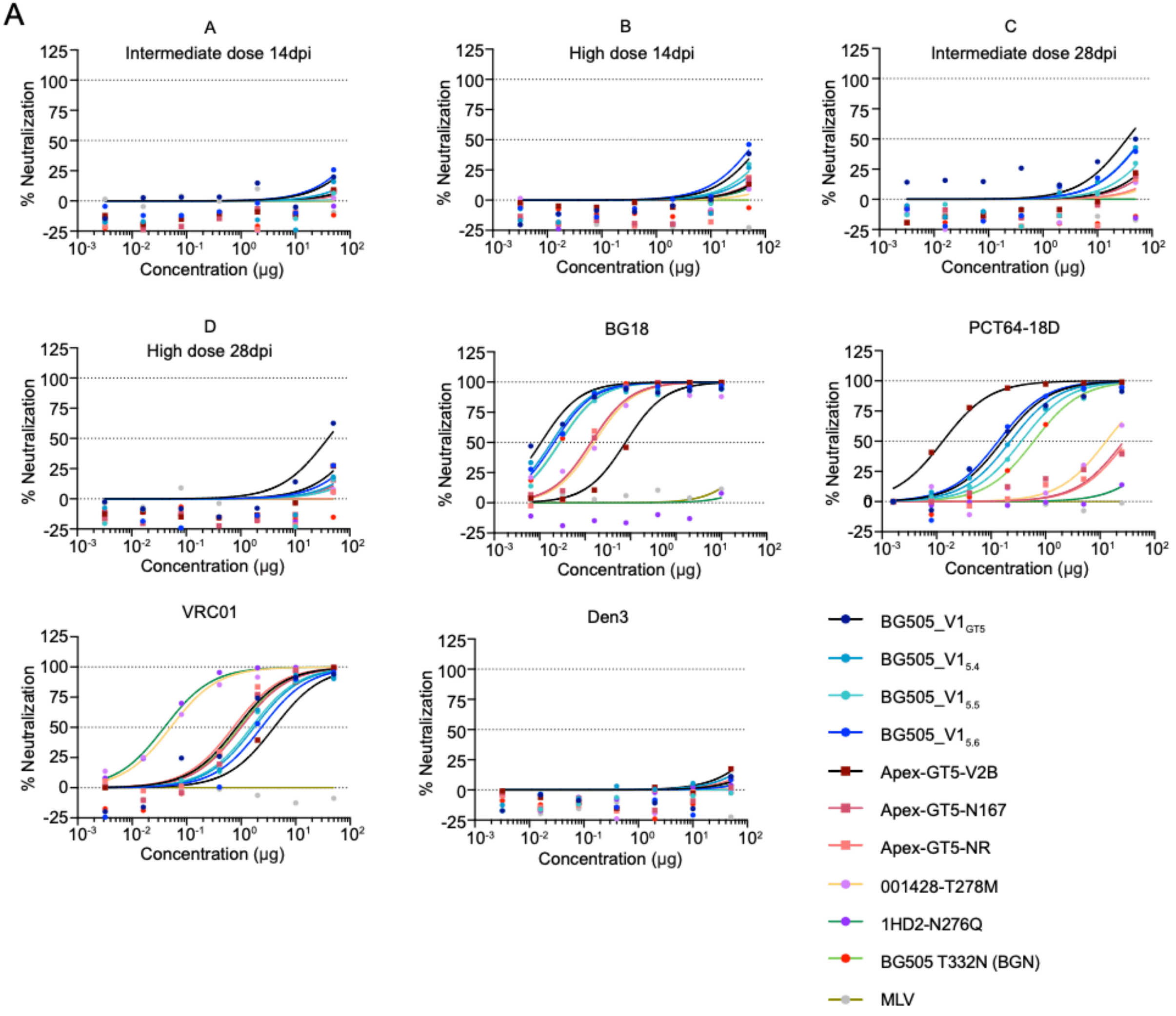

B

Viruses	A	B	C	D	BG18	PCT64	VRC01	Den3
BG505_V1$_{GT5}$	>50	>50	>50	>50	0.0017	0.1603	0.0829	>50
BG505_V1$_{5.4}$	>50	>50	>50	>50	0.0021	0.1351	0.2360	>50
BG505_V1$_{5.5}$	>50	>50	>50	>50	0.0018	0.2266	0.1728	>50
BG505_V1$_{5.6}$	>50	>50	>50	>50	0.0028	0.3592	0.1534	>50
Apex-GT5-V2B	>50	>50	>50	>50	0.0778	0.0130	0.4021	>50
Apex-GT5-N167	>50	>50	>50	>50	0.0135	>25	0.0996	>50
Apex-GT5-NR	>50	>50	>50	>50	0.0137	>25	0.0719	>50
001428-T278M	>50	>50	>50	>50	0.0159	12.9712	0.0051	>50
1HD2-N276Q	>50	>50	>50	>50	>10	>25	0.0039	>50
BG505 T332N (BGN)	>50	>50	>50	>50	0.0027	0.6071	0.0912	>50
MLV	>50	>50	>50	>50	>10	>25	>5	>50

Figure S13. Neutralization potency of murine serum, related to Figure 7.

Neutralization potency ($IC_{50,}$ μg/ml) of purified serum IgGs against native pseudoviruses. BG505_$V1_{GT5}$, BG505_$V1_{5.4}$, BG505_$V1_{5.5}$ and BG505_$V1_{5.6}$ are four variants of BG505 containing V1 loop modification as described in (*24*); Apex-GT5-V2B, Apex-GT5-N167 and Apex-GT5-NR are three variants of BG505 containing ApexGT mutations as described in (*25*). Murine leukemia virus (MLV) and Den3 bnAb against dengue virus are negative controls. A indicates serum IgGs of intermediate dose group (2 μg each) 14 dpi; B indicates serum IgGs of high dose group (10 μg each) 14 dpi; C indicates serum IgGs of intermediate dose group 28 dpi; D indicates serum IgGs of high dose group 28 dpi. Mature BG18, PCT64 and VRC01 monoclonal bnAbs are positive controls. Serum IgGs from pooled 10–12 mice in each condition for the assay.

(**A**) Neutralization plots. GraphPad Prism Non-linear regression fit. Purified serum IgG (groups A-D) and mAbs controls vs 10-virus panel. MLV virus control.

(**B**) IC50 summary table. Purified serum IgG (groups A to D) and Den3 control (IC50 >50 μg /ml), BG18 (IC50 >10 μg /ml), PCT64 (IC50 >25 μg /ml), VRC01 (IC50 >5 μg /ml).

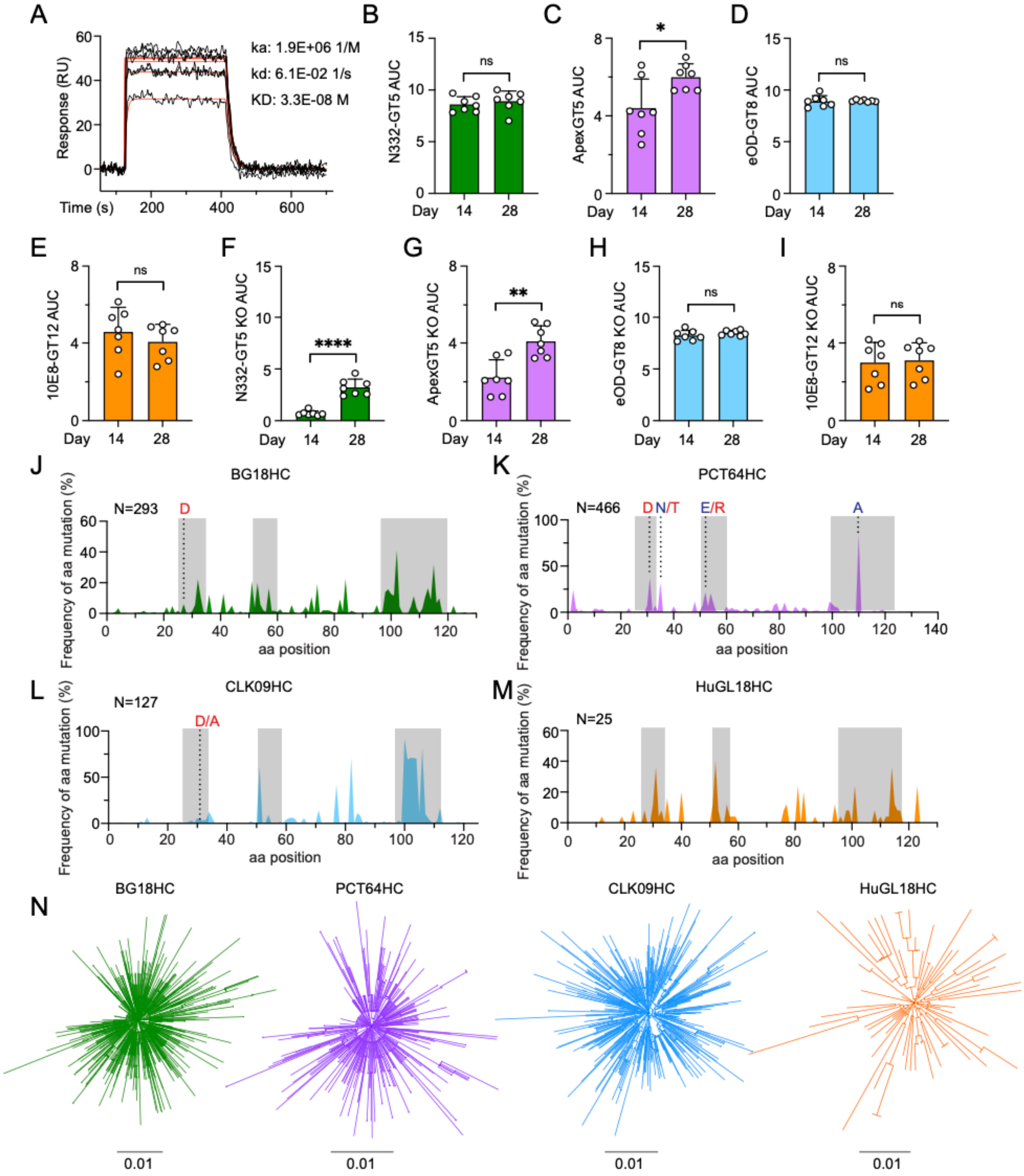

Figure S14. Simultaneous activation of four bNAb precursors elicited serum antibody responses and drove precursor SHM efficiently, related to Figure 8.

(**A**) SPR sensorgram evaluating the binding of 10E8-GT12 to HuGL18. A four-fold titration series of 10E8-GT12 starting at 50 μM was passed over HuGL18 IgG captured onto the chip surface and

data were fitted to a 1:1 binding model. Black curves represent experimental data and red curves show the best fit.

(**B to I**) ELISA quantification of serum IgG AUC values for WT proteins of N332-GT5 (B), ApexGT5 (C), eOD-GT8 (D), 10E8-GT12 (E) and epitope KO proteins of N332-GT5 (F), ApexGT5 (G), eOD-GT8 (H) and 10E8-GT12 (I) 14 and 28 dpi. Each symbol represents a different mouse. n=7 in each condition. Statistics generated using Student's t test (B and C, E and G to I) or Welch's t test (D and F). Bars represent mean + SD. Not significant (ns); **P* < 0.05; ***P* < 0.01; *****P* < 0.0001.

(**J to M**) Per site heavy chain aa mutation frequency 28 dpi. CDRs boxed in grey; key mature (red)—or, for PCT64, on-track (dark blue) (*56*)—mutations marked with letters. N at top left indicates number of sequences included in analysis. gl BG18 HC (J); LMCA PCT64 HC (K); CLK09 HC (L); $HuGL18^{H}$ HC (M).

(**N**) Phylogenetic tree plots of each lineage after quadruple GT mRNA-LNP 14 dpi. Each tree denotes a clonal lineage. Branch lengths are scaled by estimated heavy chain nt mutations. gl BG18 HC (green); LMCA PCT64 HC (pink); CLK09 HC (blue); $HuGL18^{H}$ HC (orange). Tree Scale (0.01) indicates the number of substitutions per site.

```
BG18 mature HC        QVQLRESGPGLVKPSETLSLSCTVSNDSRPSDHSWTWVRQSPGKALEWIGDIHYNGATTYNPSLR
BG18 iGL D28 HC       QVQLQESGPGLVKPSGTLSLTCAVSGDSISSSNWWTWVRQPPGKGLEWIGGIYHSGSTNYNPSLK
BG18 iGL HC           QVQLQESGPGLVKPSGTLSLTCAVSGGSISSSNWWSWVRQPPGKGLEWIGEIYHSGSTNYNPSLK

BG18 mature HC        SRVRIELDQSIPRFSLKMTSMTAADTGMYYCARNAIRIYGVVALGEWFHYGMDVWGQGTAVTVSS
BG18 iGL D28 HC       SRVTISVDQSKNQFSLKLSSVTAADTAVYYCARNAISIFGVVVLGENDYYGMDVWGQGTTVTVSS
BG18 iGL HC           SRVTISVDKSKNQFSLKLSSVTAADTAVYYCARNAITIFGVVALGEYYYYGMDVWGQGTTVTVSS

PCT64 mature HC       EVQLVESGGGLVKPGGSLRLSCVGSEFTFSDAWMTWVRQAPGKGLEWVGHMRPTPEGGAKDYAAP
PCT64LMCA D28 HC      EVQLVESGGGLVKPGGSLRLSCAASGFTFSDAWMTWVRQAPGKGLEWVGRVRSKTDGGTTDYAAP
PCT64LMCA HC          EVQLVESGGGLVKPGGSLRLSCAASGFTFSNAWMSWVRQAPGKGLEWVGRIKSKTDGGTTDYAAP

PCT64 mature HC       VKGRFTVSRDDSKRTLYLQMNSLKIEDTAVYYCMTGVEKGDFWSDDYSQHYNTYLIDVWGKGTTV
PCT64LMCA D28 HC      VKGRFTISRDDSKNTLYLQMNSLKTEDTAVYYCTTGVETYDFWSAYDDHYYDYYFRDVWGKGTTV
PCT64LMCA HC          VKGRFTISRDDSKNTLYLQMNSLKTEDTAVYYCTTGVETYDFWSGYDDHYYDYYFRDVWGKGTTV

PCT64 mature HC       TVSS
PCT64LMCA D28 HC      TVSS
PCT64LMCA HC          TVSS

N6 mature HC          RAHLVQSGTAMKKPGASVRVSCQTSGYTFTAHILFWFRQAPGRGLEWVGWIKPQYGAVNFGGGFR
Clk09 D28 HC          QVQLIQSGAEVKKPGASVKVSCKASGYTFTDYYIHWVRQAPGQGLEWMGWINPNSGGTNYAQKFQ
Clk09 gl HC           QVQLVQSGAEVKKPGASVKVSCKASGYTFTGYYMHWVRQAPGQGLEWMGWINPNSGGTNYAQKFQ

N6 mature HC          DRVTLTRDVYREIAYMDIRGLKPDDTAVYYCARDRSYGDSSWAL-DAWGQGTTVVVSA
Clk09 D28 HC          DRVTMTRDTSISTAYMELSRLRSDDTAVYYCARVGGLWFGEKKTFDMWGQGTLVTVSS
Clk09 gl HC           GRVTMTRDTSISTAYMELSRLRSDDTAVYYCARVEGLWFGEKKTFDIWGQGTMVTVSS

10E8 mature HCDR3     YYDFWSGYPP
HuGL18 D28 HCDR3      YYDFWSGLSL
HuGL18 gl HCDR3       YYDFWSGLSM
```

Figure S15. Amino acid sequence alignment of germline, mature and primed heavy chains, related to Figure 8.

BG18 inferred germline, PCT64^{LMCA}, CLK09 and HuGL18^{H} heavy chain sequences (bottom) aligned to post-prime sequences recovered at day 28 (D28, center) and their respective mature bnAb sequence—BG18, PCT64, or, in the case of CLK09, one of the mature VRC01 antibody N6 (*82*) and the key recognition motif for 10E8-class mature HCDR3s (*28*) (top). Post-prime sequences with a relatively high rate of key mutations were selected for D28 comparison. Red highlights post-prime mutations found in the mature bnAb and identified as key to function (note: Asp (D) at the 31 site is shared by some VRC01 mature bnAbs, while N6 and some others have an Ala (A) at that site); orange highlights post-prime mutations with identity to the mature bnAbs where a key role in binding has not been proposed; blue highlights “on-track” mutations from the pre-mature lineage,

and is used only for PCT64 (*25*, *26*); yellow highlights all other mutations acquired post-prime; grey denotes aa in post-prime or inferred germline sequences which differ from the mature bnAbs.

Supplemental Table 1. Key resources. Reagents by type associated with supplier and catalogue number.

**Antibodies**	**Supplier**	**Cat number**
BV786-B220	BD Bioscience	563894
BV510-B220	BD Bioscience	563103
Alexa Fluor 700-Ly6G/Ly-6C (Gr-1)	BioLegend	108422
APC-eFluor™ 780-Ly6G/Ly-6C (Gr-1)	eBioscience	2689187
Alexa Fluor 700-CD3	BioLegend	100216
APC-eFluor™ 780-CD3	eBioscience	2611862
Alexa Fluor 700-F4/80	BioLegend	123130
APC-eFluor™ 780-F4/80	eBioscience	2611773
BV510-CD38	BD Bioscience	740129
PE-Cy7-CD95	BD Bioscience	557653
PerCP Cy5.5-CD45.1	BioLegend	110728
PE-CD45.2	BioLegend	109808
BV786-CD45.2	BD Bioscience	563686
APC-Cy7-IgM	BioLegend	406516
PE-Cy7-IgD	BioLegend	405720
BV650-IgD	BioLegend	405721
PE/Dazzle™ 594-CD138	BioLegend	142528
**Commercial chemicals**		
LIVE/DEAD™ Fixable Blue Dead Cell Stain Kit	Invitrogen	L23105
4',6-diamidino-2-phenylindole, dihydrochloride (DAPI)	Invitrogen	D1306
Mouse BD Fc Block™	BD Bioscience	553142
Alexa Fluor 647-Streptavidin	BioLegend	405237
BV421- Streptavidin	BD Bioscience	563259
Alexa Fluor 488-Streptavidin	BioLegend	405235
Alexa Fluor 594-Streptavidin	BioLegend	405240
BV605-Streptavidin	BioLegend	405229
BUV395-Streptavidin	BD Bioscience	564176
Sigma Adjuvant	Sigma	S6322
Dulbecco's Phosphate-Buffered Saline (DPBS)	Corning	21-031-CV
Fetal Bovine Serum (FBS)	Sigma	F4135
ACK lysing buffer	Lonza	BP10-548E
Pan B Cell Isolation Kit II, mouse	Miltenyi	130-104-443
LS Columns	Miltenyi	130-042-401
His-tag Antibody, pAb, Rabbit	GenScript	A00174
Bovine Serum Albumin (BSA)	Sigma	A4612
Sodium hydroxide	Sigma	S5881
Alkaline Phosphatase AffiniPure™ Goat Anti-Mouse IgG, Fcγ fragment specific	Jackson ImmunoResearch	115-055-071
SIGMA*FAST*™ p-Nitrophenyl phosphate Tablets	Sigma	N2770
Uranyl Formate	EMS	22450

Papain	Sigma-Aldrich	P4762
Protein G Sepharose	Cytiva	17061805
TotalSeq™-C0301 anti-mouse Hashtag 1 Antibody	BioLegend	155861
TotalSeq™-C0302 anti-mouse Hashtag 2 Antibody	BioLegend	155863
TotalSeq™-C0303 anti-mouse Hashtag 3 Antibody	BioLegend	155865
TotalSeq™-C0304 anti-mouse Hashtag 4 Antibody	BioLegend	155867
TotalSeq™-C0305 anti-mouse Hashtag 5 Antibody	BioLegend	155869
TotalSeq™-C0306 anti-mouse Hashtag 6 Antibody	BioLegend	155871
TotalSeq™-C0307 anti-mouse Hashtag 7 Antibody	BioLegend	155873
TotalSeq™-C0308 anti-mouse Hashtag 8 Antibody	BioLegend	155875
TotalSeq™-C0309 anti-mouse Hashtag 9 Antibody	BioLegend	155877
TotalSeq™-C0310 anti-mouse Hashtag 10 Antibody	BioLegend	155879
TotalSeq™-C0311 anti-mouse Hashtag 11 Antibody	BioLegend	155881
TotalSeq™-C0312 anti-mouse Hashtag 12 Antibody	BioLegend	155883
TotalSeq™-C0313 anti-mouse Hashtag 13 Antibody	BioLegend	155885
TotalSeq™-C0314 anti-mouse Hashtag 14 Antibody	BioLegend	155887
TotalSeq™-C0315 anti-mouse Hashtag 15 Antibody	BioLegend	155889
TotalSeq™-C0316 anti-mouse Hashtag 16 Antibody	BioLegend	155891
TotalSeq™-C0317 anti-mouse Hashtag 17 Antibody	BioLegend	113931
TotalSeq™-C0318 anti-mouse Hashtag 18 Antibody	BioLegend	113929
TotalSeq™-C0325 anti-mouse Hashtag 19 Antibody	BioLegend	155893
TotalSeq™-C0326 anti-mouse Hashtag 20 Antibody	BioLegend	155895
Chromium Next GEM Single Cell 5′ Kit v2	10x Genomics	1000263
Library Construction Kit	10x Genomics	1000352
Chromium Single Cell Mouse BCR Amplification Kit	10x Genomics	1000255
Chromium Next GEM Chip K Single Cell Kit	10x Genomics	1000286
Dual Index Kit TT Set A, 96 rxns	10x Genomics	1000215
Dual Index Kit TN Set A, 96 rxns	10x Genomics	1000250
**Reagents generated in lab or Moderna**		
N332-GT5 His-tagged trimer protein	Schief lab	N/A
N332-GT5 KO His-tagged trimer protein	Schief lab	N/A
N332-GT5 His-Avi-tagged trimer protein	Schief lab	N/A
N332-GT5 KO His-Avi-tagged trimer protein	Schief lab	N/A
ApexGT5 His-tagged trimer protein	Schief lab	N/A
ApexGT5 His-Avi-tagged trimer protein	Schief lab	N/A
ApexGT5 KO His-Avi-tagged trimer protein	Schief lab	N/A
ApexGT6 His-Avi-tagged trimer protein	Schief lab	N/A
eOD-GT8 60mer protein	Schief lab	N/A
eOD-GT8 His-tagged protein	Schief lab	N/A
eOD-GT8 KO His-tagged protein	Schief lab	N/A
eOD-GT8 His-Avi-tagged protein	Schief lab	N/A
eOD-GT8 KO His-Avi-tagged protein	Schief lab	N/A
10E8-GT12 His-tagged protein	Schief lab	N/A
10E8-GT12 KO His-tagged protein	Schief lab	N/A

10E8-GT12 His-Avi-tagged protein	Schief lab	N/A
10E8-GT12 KO His-Avi-tagged protein	Schief lab	N/A
Membrane-bound N332-GT5 trimer mRNA-LNP	Moderna	N/A
Membrane-bound ApexGT5 trimer mRNA-LNP	Moderna	N/A
Soluble ApexGT6 trimer mRNA-LNP	Moderna	N/A
Membrane-bound ApexGT6 trimer mRNA-LNP	Moderna	N/A
Soluble eOD-GT8 60mer mRNA-LNP	Moderna	N/A
Soluble 10E8-GT12 24mer mRNA-LNP	Moderna	N/A
